# Relationship between hepatic and mitochondrial ceramides: a novel in vivo method to track ceramide synthesis

**DOI:** 10.1016/j.jlr.2023.100366

**Published:** 2023-04-05

**Authors:** Justine M. Mucinski, Jonas M. McCaffrey, R. Scott Rector, Takhar Kasumov, Elizabeth J. Parks

**Affiliations:** 1 Department of Nutrition and Exercise Physiology, University of Missouri, Columbia, Missouri, USA; 2 Division of Gastroenterology and Hepatology, Department of Medicine, School of Medicine, University of Missouri, Columbia, Missouri, USA; 3 Research Service, Harry S Truman Memorial Veterans Medical Center, Columbia, Missouri, USA; 4 Department of Pharmaceutical Sciences, College of Pharmacy, Northeast Ohio Medical University, Rootstown, Ohio, USA

**Keywords:** ceramides, kinetics, lipidomics, liver, mitochondria, stable isotope tracers

## Abstract

Ceramides (CERs) are key intermediate sphingolipids implicated in contributing to mitochondrial dysfunction and the development of multiple metabolic conditions. Despite the growing evidence of CER role in disease risk, kinetic methods to measure CER turnover are lacking, particularly using in vivo models. The utility of orally administered ^13^C_3_, ^15^N l-serine, dissolved in drinking water, was tested to quantify CER 18:1/16:0 synthesis in 10-week-old male and female C57Bl/6 mice. To generate isotopic labeling curves, animals consumed either a control diet or high-fat diet (HFD; *n* = 24/diet) for 2 weeks and varied in the duration of the consumption of serine-labeled water (0, 1, 2, 4, 7, or 12 days; *n* = 4 animals/day/diet). Unlabeled and labeled hepatic and mitochondrial CERs were quantified using liquid chromatography tandem MS. Total hepatic CER content did not differ between the two diet groups, whereas total mitochondrial CERs increased with HFD feeding (60%, *P* < 0.001). Within hepatic and mitochondrial pools, HFD induced greater saturated CER concentrations (*P* < 0.05) and significantly elevated absolute turnover of 16:0 mitochondrial CER (mitochondria: 59%, *P* < 0.001 vs. liver: 15%, *P* = 0.256). The data suggest cellular redistribution of CERs because of the HFD. These data demonstrate that a 2-week HFD alters the turnover and content of mitochondrial CERs. Given the growing data on CERs contributing to hepatic mitochondrial dysfunction and the progression of multiple metabolic diseases, this method may now be used to investigate how CER turnover is altered in these conditions.

Ceramides (CERs) serve as a central hub in sphingolipid metabolism and provide precursors for the production of more complex lipid molecules (e.g., sphingomyelin). The de novo synthesis pathway occurs primarily in the endoplasmic reticulum (1, 2) and begins with the condensation of l-serine and, most often, with the 16-carbon FA (16:0, palmitate) through the action of the enzyme serine palmitoyl transferase (SPT) (3). The addition of a second FA is catalyzed by one of six ceramide synthases (CERS 1–6), each of which have specificity for FA of varying chain length (4). In a final step of synthesis, the 4–5 carbon bond in the backbone is desaturated by dihydroceramide desaturase (DEGS1), thereby converting dihydroCER (dhCER) to CER. Recently, CER synthetic enzymes were identified within mitochondrial membranes for the first time (5, 6). Mitochondrial CERs (7, 8, 9) are implicated in decreased mitochondrial respiration and tissue function (9, 10, 11, 12, 13) and increased cellular reactive oxygen species and apoptosis (8, 12, 14, 15). In addition, plasma and tissue CER concentrations are associated with metabolic dysfunction (12, 16, 17, 18, 19, 20, 21, 22, 23, 24) and increased cardiovascular risk (24, 25, 26, 27), with specific species (e.g., CER with 16:0 or 18:0 in the fatty acyl site) conferring greater cardiometabolic risk than others (e.g., 24:1) (8, 9, 12, 13, 14). Indeed, CER 16:0 has garnered particular interest because of its role in predicting CVD and diabetes risk in large population-based studies (28, 29) and its mechanistic involvement in insulin resistance, mitochondrial function, and apoptosis as demonstrated in basic knockout studies (8, 12, 30). While the role of CER in metabolic and cardiovascular dysfunction has been studied extensively, limited methods are available to measure CER kinetics. Ultimately, in addition to excess CER concentrations, CER turnover may be more mechanistically related to the toxicity of this lipid, and kinetic assays are important in the development of therapies to lower concentrations and reduced disease burden.

With advances in MS sensitivity, methods for isotopic labeling of CER synthesis have recently been published (31, 32). In cell culture and rodent models, kinetic studies using stable isotopes (13, 33, 34, 35, 36, 37, 38, 39, 40, 41, 42, 43) and non-naturally occurring odd-chain analogs (44, 45) are available, and in humans, CER synthesis in skeletal muscle has been measured via isotope labeling (46, 47, 48). Isotopically labeled serine and/or palmitate can be incorporated into the backbone of the sphingolipid through the SPT enzymatic reaction, whereas palmitate can also be incorporated into the fatty acyl chain through CERS5 and CERS6. Labeled palmitate has been used in rat models for hepatic CER kinetics (34, 35), whereas only one study has used labeled water (D_2_O) or serine to estimate plasma CER kinetics in mice (33). No study has investigated the use of an oral serine isotope to quantify hepatic and mitochondrial CER kinetics or has compared hepatic mitochondrial CER content or synthesis to whole liver tissue content or synthesis.

The aim of the current study was to develop the use of a stable isotope, ^13^C_3_, ^15^N l-serine, delivered orally, to quantify CER 16:0 production within whole liver and isolated hepatic mitochondria. CER 16:0 was specifically targeted because of strong data implicating this species in cardiovascular and metabolic diseases (19, 24, 28, 29) and in mitochondrial dysfunction (8, 11, 12, 15, 30). C57Bl/6 mice were fed a control diet (CD; low-fat) or high-fat diet (HFD) for 2 weeks. We hypothesized that short-term HFD feeding would induce elevated total liver CER concentrations, which would be associated with elevated turnover rates. Surprisingly, only mitochondrial CER content and turnover was shifted with high-fat feeding. Our results support the ease of use of serine in quantifying 16:0 CER synthetic flux in hepatic tissue and mitochondria, and these methods may be adapted for use in other tissues/organelles, CER species, and experimental models.

## Materials and methods

### Study design

**Fig. 1:**
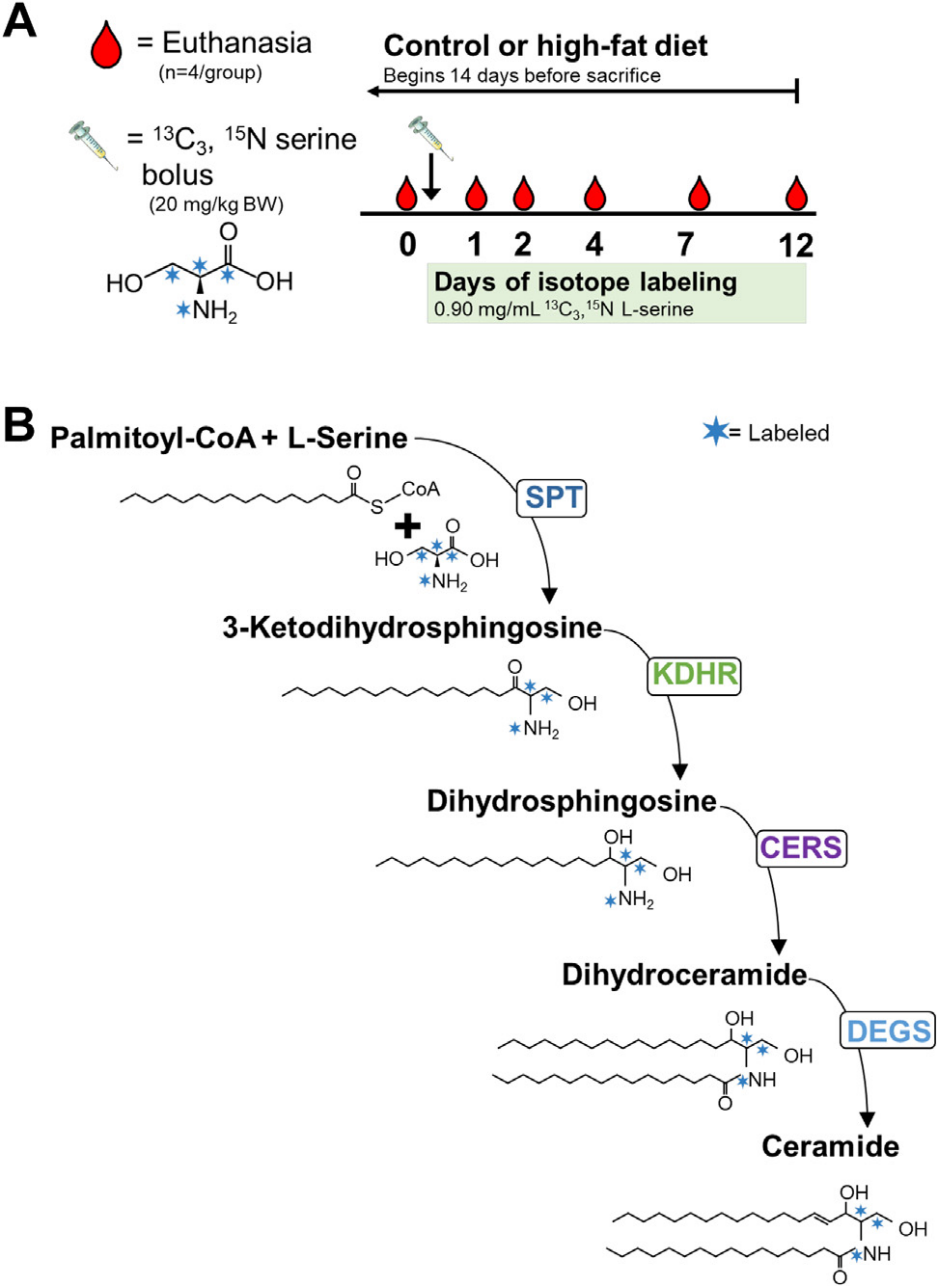
Study design and incorporation of ^13^C_3_, ^15^N l-serine into ceramide. Blue stars indicate a ^13^C or ^15^N isotope. A: Study design: Animal euthanized on day 0 received no isotope—either by IP bolus or in the drinking water. B: Incorporation of the serine label into ceramide through the de novo synthesis pathway. DEGS, dihydroceramide desaturase; KDHR, 3-ketodihydrosphingosine reductase.

Following a 2-week acclimatization period, 10-week-old male (*n* = 24) and female (*n* = 24) C57Bl/6 mice were switched from chow to either a CD (Research Diets, Inc; catalog no.: D12450J) or HFD (Research Diets, Inc; catalog no.: D12492). The formulated CD contained 20% of energy (%E) from protein, 10%E from fat, and 70%E from carbohydrate (7%E from sucrose). The HFD contained 20%E from protein, 60%E from fat, and 20%E from carbohydrate (7%E from sucrose). As shown in Fig. 1A, each animal remained on the assigned diet for 2 weeks. Both before the start of the diet and on the day before euthanasia, body composition was measured via echoMRI (4 in 1–1,100 analyzer, EchoMRI®). The animals received an oral serine isotope (^13^C_3_,^15^N l-serine; Cambridge Isotope Laboratories, Andover, MA) to label the backbone of de novo synthesized CERs (labeling pattern shown in Fig. 1B). A bolus of the isotope was delivered by intraperitoneal injection (20 mg/kg BW) on the first day of labeling and then in the drinking water (0.90 mg/ml) until the day of euthanasia. To obtain CER kinetics, four mice/group (two males and two females) were euthanized on days 0, 1, 2, 4, 7, and 12 after administration of the tracer. Animals euthanized on day 0 received no label and were included to measure background labeling of CER 16:0. CERs and dhCERs were isolated from both total liver tissue and from hepatic mitochondria and analyzed by HPLC–tandem MS with multiple reaction monitoring (MRM), as described later.

### Animal care and terminal procedures

Animals were obtained from Jackson Laboratories and housed two per cage with a light cycle from 0700 to 1,900 h in constant room temperature of 21–22°C. On the day of euthanasia, food was removed, and mice fasted for 5 h (0500–1,000, with continuous access to water) before being anesthetized with sodium pentobarbital (80–100 mg/kg; intraperitoneal injection). At terminal experiments, blood was obtained through intra-aortic puncture, serum samples were divided into aliquots for quantification of metabolites (glucose, insulin, triacylglycerols [TGs], and FFAs), and animals were euthanized via removal of the heart. Livers were quickly excised, weighed, and aliquots were *1*) placed in an ice-cold isolation buffer for mitochondrial isolation and *2*) flash frozen in liquid nitrogen to be stored at −80°C for later processing. All animals included in this study were cared for in accordance with National Institute of Health guidelines, and the protocol was approved in advance by the University of Missouri IACUC (protocol #9832).

### Mitochondria isolation

Hepatic mitochondria were isolated as previously described (49). Briefly, once excised and weighed, approximately 333 ± 58 mg of liver tissue was minced and gently homogenized using a smooth surface probe in mitochondrial isolation buffer A (220 mM mannitol, 70 mM sucrose, 10 mM Tris-base, 1 mM EDTA; pH 7.4) (49). The sample was centrifuged at 1,500 *g* for 10 min at 4°C, the pellet discarded, and the supernatant underwent three additional centrifugations (6,000–8,000 *g*) where the resultant pellet was retained and resuspended (in buffer A or in A with 0.1% bovine serum albumin) with gentle glass-on-glass homogenizations. The final pellet was homogenized with 400 μl of phosphate-buffered saline (∼1:1 starting w/v). The sample was stored at −80°C and used for protein determination via BCA assay (catalog no.: 23225; ThermoFisher Scientific), CER extraction, selected Western blots, and citrate synthase activity assays. Mitochondrial citrate synthase activity, an established surrogate of mitochondrial content, was measured as previously described (50).

### CER concentrations and enrichment

CERs were extracted from liver homogenate and hepatic mitochondria. Briefly, for total liver CER, ∼10 mg of frozen tissue was weighed and homogenized in buffer B (200 μl, sucrose 250 mM, KCl 25 mM, Tris-base 50 mM, and EDTA 0.5 mM). Samples (equivalent to 1.2 ± 0.1 mg of total protein) and calibration curves were spiked with 50 μl of the internal standard C18:1/17:0 (50 ng), and CERs were extracted according to the protocol of Bligh and Dyer (51). The organic phase was removed and dried under nitrogen gas, and samples were stored in −80°C until LC/MS analysis. For mitochondrial CERs, mitochondria isolated from ∼150 mg fresh tissue (200 μl of mitochondrial isolate in PBS, 1.5 ± 0.2 mg of protein) was extracted as described for liver tissue. Both liver and mitochondria samples were analyzed using the same LC/MS method, and CERs were quantified using HPLC–tandem MS ESI in positive ion scanning mode (52). Standards and samples were dissolved in 100 μl of 0.1% formic acid solution in methanol-water (85:15) and then injected into a Waters HPLC device (2690 Separation Module, Milford, MA) and separated through a Vydac® 200MS™ C8 column (2.1 × 100 mm, 5 μm, P.J. Cobert Associates, St. Louis, MO). CERs were analyzed using MRM scanning for each molecular ion with the mass to charge ratio (*m/z*) 264 daughter ion across all species (*m/z* 266 for dhCER). Only targeted CERs and dhCER with these daughter ions were monitored; thus, this analysis did not include quantification of sphingoid bases with alternative chain lengths or degree of saturation. Individual unlabeled CER species and the labeled 16:0 CER MRM transitions and retention times are listed in Supplemental Table S1. Calibration curves used to calculate absolute concentrations included CER 18:1/14:0, 18:1/16:0, 18:1/18:0, 18:1/18:1, 18:1/20:0, 18:1/22:0, 18:1/24:0, and 18:1/24:1 and dhCER 18:0/16:0, 18:0/18:0, 18:0/24:0, and 18:0/24:1 using CER 18:1/17:0 as an internal standard. Chromatograms were analyzed using Xcalibur™ (Thermo Scientific™ 3.0.63). Assay precision was tested using a pooled liver or pooled mitochondrial sample, each of which was a combination of samples from 48 mice. To measure intra-assay variability, one pooled liver or mitochondrial sample was analyzed five times for comparison and for interassay variability, pooled liver and mitochondria samples were extracted on five separate occasions over a 2-week period and each extraction analyzed separately then compared.

Percent enrichment (%E) was calculated as the area under the peak for the labeled CER 16:0 fragment (M3, *m/z* 267) divided by the sum of the area under the peaks for total label and unlabeled (M0, M1, and M3) 16:0 CER fragments. As described in the supplemental data section (supplemental Figs. S1 and S2 and Supplemental Tables S2 and S3), an additional calculation was performed that included the use of a mathematical model based on increasing M3/M1 ratios to predict the fraction of the CER pool made new. The observed percentage of new CER from day 12 animals was measured to be 1.9 ± 0.1%, whereas the model described in the supplemental section (using the M3/M1 ratio) predicted that 2.0 ± 0.1% would have been made new. Because the natural abundance of M3 in the fragment is so low (0.083%), the vast majority of M3 observed in the animals was due to incorporation of the label during CER synthesis. The addition of M1 did not improve the calculation.

Enrichment of free hepatic l-serine was analyzed as previously described (53). In brief, ∼25 mg of liver tissue was homogenized in buffer B with 6% formic acid and eluted using an ion exchange column (50WX8-400, hydrogen form). Retained amino acids were released using ammonium hydroxide, and the eluate dried under nitrogen gas. Samples were derivatized using 100 μl Bis(trimethylsilyl) trifluoroacetamide 10% trimethylchlorosilane and analyzed by GC/MS as described (53). Briefly, l-serine enrichments were determined using electron impact ionization (70 eV) and selected ion monitoring (204–207 *m/z*) on a 6890 N gas chromatography coupled to a 5975 MS detector (Agilent Technologies, Palo Alto, CA) using a DB-17MS capillary column (30 m length, inner diameter 0.25 mm, and 0.25 μm film, part no.: 122-4732, Agilent J&W GC Columns, ChromTech, Inc, Apple Valley, MN).

### Protein quantification and enzymatic assays

To assess whether differences in CER content and turnover were driven by changes in protein content, key proteins involved in CER synthesis were measured via Western blot in whole liver homogenate and selected CER synthases in isolated mitochondria. A list of primary and secondary antibodies is presented in the supplemental data. Total protein was assessed with amido black (0.1%; MilliporeSigma) to control for differences in protein loading and transfer as previously described (50). Liver-TG content was quantified by enzymatic assay using TG (catalog no.: T2449; Sigma) and free glycerol reagent (catalog no.: F6428; Sigma) following Folch lipid extraction (50). Serum glucose (catalog no.: 997-03001; Wako), TG (same reagents listed for liver-TG), and FFA (catalog nos.: 999-34691; 995-34791; 994-02891; 990-02991, Wako) were quantified using commercially available enzymatic reagents. Serum insulin concentrations were determined using ELISA (EZRMI-13K; MilliporeSigma).

### Statistical analysis and calculations

Calculations were performed using Microsoft Excel (2016; Redmond, WA) and statistical analysis using R (version 4.1.3) and R studio (Boston, MA). Tissue and mitochondrial CER turnover curves were generated as follows: for each diet, four curves were generated. The average of four mice per group represented a single data point within a labeling curve; however, to calculate fractional and absolute synthetic rates (ASRs), individual mice (within a sex) were matched across days to produce four individual curves per diet group. Unpaired two-tailed *t*-tests were used to compare static outcomes between diets. Pearson’s R was used to quantify linear relationships between continuous variables. Data varying across time were reported as mean ± SEM, whereas static variables were reported as a mean ± SD. In Table 1, characteristics collected only at terminal (final) procedures were compared between groups using an unpaired one-tailed *t-*test because we hypothesized that liver-TG would increase with HFD feeding. Significance was set at *P* < 0.05, and *P* < 0.10 was reported as a trend. Fractional synthetic rates (FSRs) of de novo 16:0 CER (pools/day or *k*) were calculated by fitting single exponential curves (equation: y = A∞ ∗ [1−e^−*k*^*∗*^t^]; y = %E, A∞ = predicted asymptote, *k* = fractional synthesis, and *t* = time in days) to the enrichment curves in SigmaPlot (Systat Software, Inc) (54). ASRs (pmol/mg tissue/day) were calculated as the product of *k* and the absolute pool size (pmol/mg tissue). Half-lives (*t*½, day) were calculated as the natural log of two divided by fractional synthesis.

**Table 1:** Animal characteristics before and after 2 weeks of CD or HFD

Characteristic	CD		HFD		*P*
	Baseline	Final	Baseline	Final	ANOVA^a^
Body weight (g)	23.1 ± 3.4	23.4 ± 3.4	23.4 ± 3.6	27.1 ± 4.7	<0.001
Body fat (%)	11 ± 3%	13 ± 4%	11 ± 2%	23 ± 4%	<0.001

	Final	Final	*t-*Test^b^
Food intake (g/day)	2.4 ± 0.3	2.5 ± 0.2	0.116
Liver weight (mg)	947 ± 176	972 ± 191	0.321
Liver TG content (mg/g)	17.7 ± 5.7	20.9 ± 5.5	< 0.001
Serum TG (mmol/l)	0.39 ± 0.09	0.29 ± 0.08	< 0.001
Serum FFA (mmol/l)	0.26 ± 0.04	0.31 ± 0.05	0.002
Serum glucose (mmol/l)	11.7 ± 3.0	13.7 ± 2.4	0.007
Serum insulin (μU/ml)	38.0 ± 15.5	40.9 ± 18.2	0.287

Data are presented as mean ± SD; *n* = 24/group (males and females combined); baseline values are pre-diet, whereas final values were collected at 2 weeks, on the day of euthanasia.

a For body weight and percent fat, repeated-measures ANOVA was used for comparison across time between diet groups.

b Characteristics collected only at terminal (final) procedures were compared between groups using an unpaired one-tailed *t*-test.

## Results

### Animal characteristics

Body weight was similar in all animals at the start of the diet (Table 1) with males weighing ∼23–25% more than females (data not shown). Following the 2-week diet, HFD-fed animals had significantly greater total body weight and fat mass than CD animals, whereas both groups had similar liver weights and daily food intakes (Table 1). At the end of 2 weeks, animals were different in their neither average food intake nor total liver weight. However, animals consuming HFD had significantly elevated liver-TG (*P* < 0.001) and lower circulating TG (*P* < 0.001). Serum FFA (*P* = 0.002) and glucose (*P* = 0.007) were significantly higher, whereas serum insulin concentrations were not different (*P* = 0.287).

### Liver and mitochondrial CER and dhCER content

**Fig. 2:**
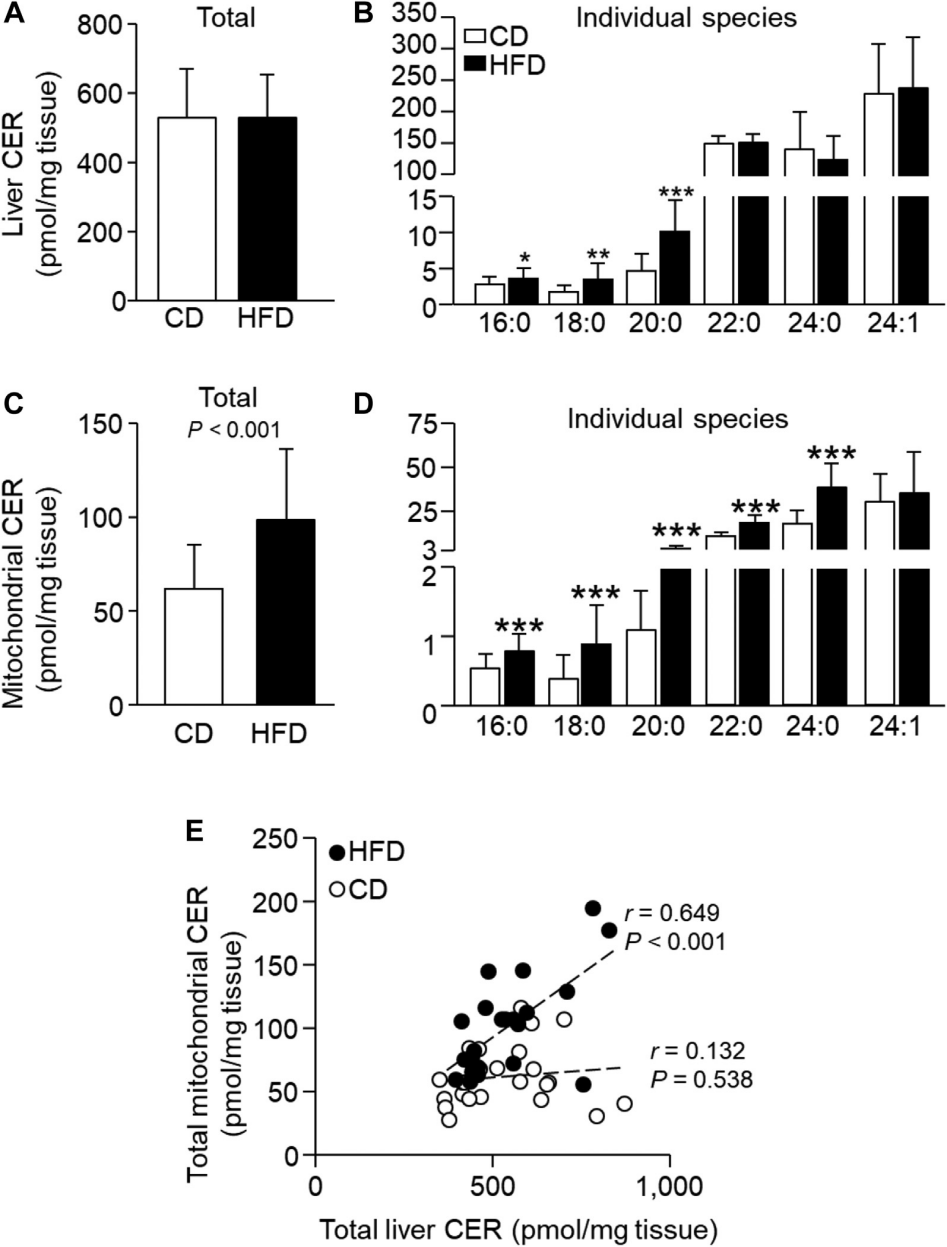
Whole liver homogenate and isolated hepatic mitochondrial ceramide concentrations. Data are presented as mean ± SD; *n* = 24/diet. Unpaired two-tailed *t*-tests. CD (white bars) versus HFD (black bars) within total or individual species: #*P* < 0.10, ∗*P* < 0.05, ∗∗*P* < 0.01, ∗∗∗*P* < 0.001. All CER presented contain an 18:1 backbone. A and B: Total or individual whole liver homogenate CER species. C and D: Total or individual isolated hepatic mitochondrial CER species. E: Relationship between whole liver homogenate and isolated hepatic mitochondrial CER (Pearson correlation).

The MRM analytical technique distinguished between CERs and dhCERs with the same fatty acyl chain (e.g., CER 16:0 and dhCER 16:0) because of the combined analysis of parent to daughter ion transitions and retention times, which differ for CERs and dhCERs (Supplemental Table S1). Based on the internal standard and the calibration curves, absolute concentrations were calculated in units of pmol/mg wet weight of liver tissue. Contrary to the hypothesis, the short-term HFD did not alter total liver CER concentrations (Fig. 2A). However, the saturated hepatic CER species 16:0, 18:0, and 20:0 were significantly greater in HFD-fed animals (30%, 100%, and 115%, respectively; Fig. 2B). By contrast, for total mitochondrial CERs, concentrations were significantly greater with HFD feeding (Fig. 2C), and this observation was characterized by increases in the individual mitochondrial species 16:0 (46%), 18:0 (128%), 20:0 (255%), 22:0 (68%), and 24:0 (116%) (Fig. 2D). In a comprehensive review of the published literature, we were not able to find data on the relationship between total liver and hepatic mitochondrial CER contents. When all animals were analyzed together, a significant positive relationship was found between the two pools (*r* = 0.360, *P* = 0.012); however, this relationship was driven solely by the HFD animals (Fig. 2E). One interpretation of these data is that the HFD elicited a shift in hepatocellular CER storage, favoring the mitochondrial pool. Indeed, HFD-fed animals had a greater proportion of total liver CER within the mitochondrial pool than CD-fed animals (19 ± 5% vs. 12 ± 4%, respectively; *P* < 0.0001, supplemental Fig. S3). With regard to sex as a biological variable (Supplemental Table S4), a significant main effect of sex was observed for total hepatic and mitochondrial CERs. Within both pools, 18:0 and 20:0 concentrations were significantly different across sex and diet (*P* < 0.05, interaction term) with the greatest concentrations found in female mice consuming the HFD.

Total liver dhCER concentrations did not differ between the diet groups (supplemental Fig. S4A), whereas HFD animals exhibited significantly lower dhCER 24:0 (32%, supplemental Fig. S4B). By contrast, total mitochondrial dhCER concentrations were greater in HFD animals with significantly greater 18:0/16:0 (50%), 18:0/18:0 (156%), and 18:0/24:0 (158%, supplemental Fig. S4, C and D). Like CERs, a positive relationship was observed between total liver and mitochondrial dhCERs only in HFD-fed animals (supplemental Fig. S4E). Analysis of the interassay and intra-assay variability demonstrated high precision between sample extractions and within the analytical method (Table 2). Specifically, interassay coefficient of variations were between 0.5% and 5.4% for both hepatic CER pools (liver and isolated mitochondria) and 0.6% and 7.8% coefficient of variation for the intra-assay precision. These values agree with a previous report using the similar methodology (52).

**Table 2:** Interassay and intra-assay precision of LC/MS CER assay for whole liver homogenate and isolated hepatic mitochondrial CERs

Liver ceramide	Interassay (%)	Intra-assay (%)
*18:1/14:0*	0.8	1.0
*18:1/16:0*	3.5	4.4
*18:1/18:0*	0.5	0.6
*18:1/18:1*	4.5	3.6
*18:1/20:0*	3.7	4.3
*18:1/22:0*	0.5	1.4
*18:1/24:0*	2.1	3.6
*18:1/24:1*	2.3	4.4

Mitochondrial ceramide	Interassay	Intra-assay
*18:1/14:0*	1.7	1.6
*18:1/16:0*	5.0	2.9
*18:1/18:0*	2.1	4.3
*18:1/18:1*	5.4	3.7
*18:1/20:0*	3.6	5.8
*18:1/22:0*	2.6	5.9
*18:1/24:0*	4.4	7.8
*18:1/24:1*	3.6	4.3

Data are presented as the coefficient of variation.

Interassay: A single pooled sample (whole liver homogenate or isolated hepatic mitochondria was pooled from *n* = 48 mice) was extracted and analyzed five times.

Intra-assay: A pooled sample (whole liver homogenate or isolated hepatic mitochondria was pooled from *n* = 48 mice) was extracted five times on different days across 2 weeks, and each extraction was analyzed once.

### CER 16:0 turnover

**Fig. 3:**
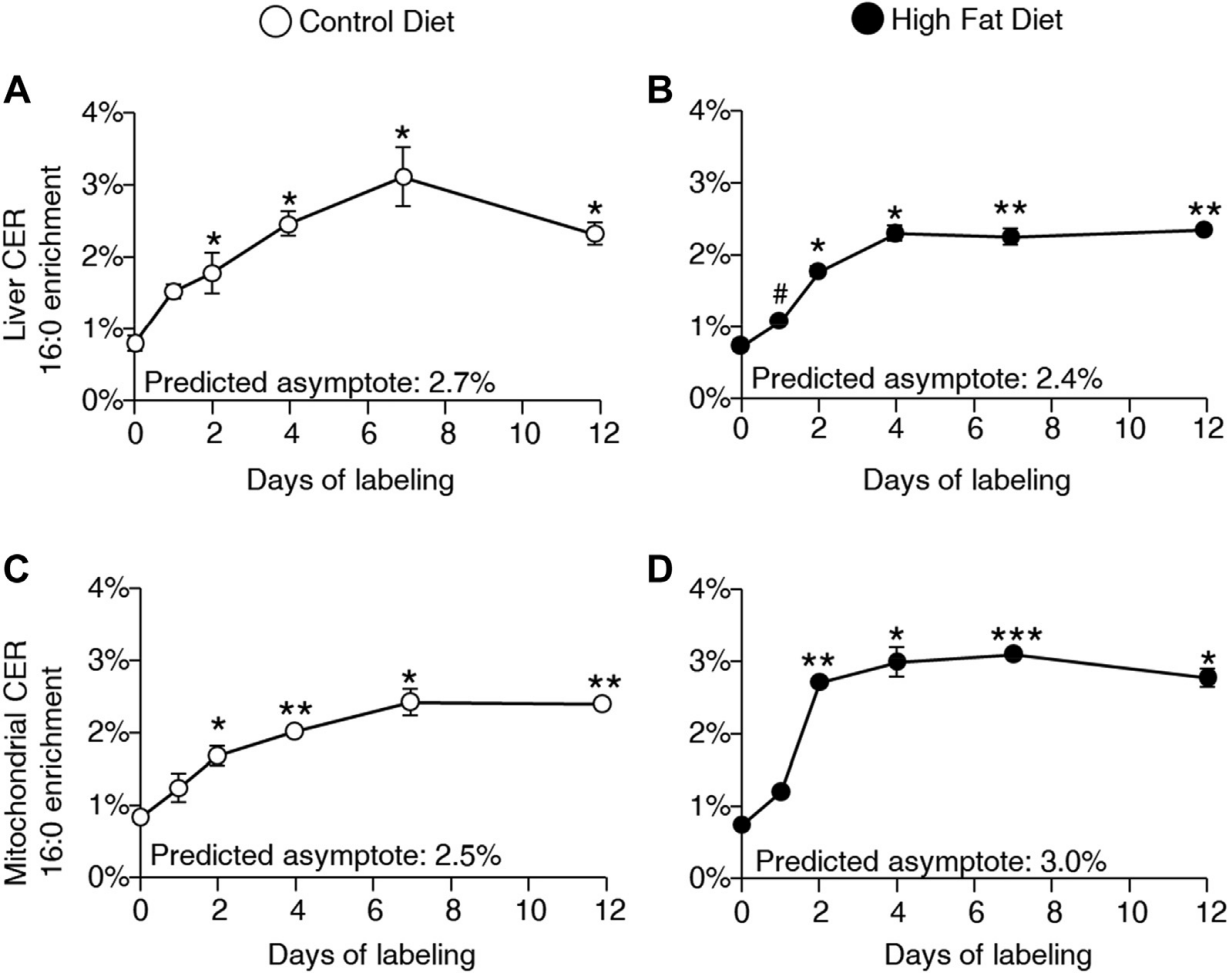
^13^C_3_, ^15^N l-serine labeling profile of whole liver homogenate and isolated hepatic mitochondrial 16:0 CER. Data are presented as mean ± SEM in open circles for CD and filled circles for HFD (*n* = 2–4 mice/time point). ANOVA for differences across labeling days (all *P* < 0.0001) and post hoc *t*-test with Bonferroni adjustments #*P* < 0.10, ∗*P* < 0.05, ∗∗*P* < 0.01, ∗∗∗*P* < 0.001 versus unlabeled (day 0). A and B: Whole liver homogenate CER 16:0 percent enrichment across labeling days (0, 1, 2, 4, 7, and 12) for CD and HFD. C and D: Isolated hepatic mitochondrial CER 16:0 percent enrichment across labeling days (0, 1, 2, 4, 7, and 12) for CD and HFD.

**Fig. 4:**
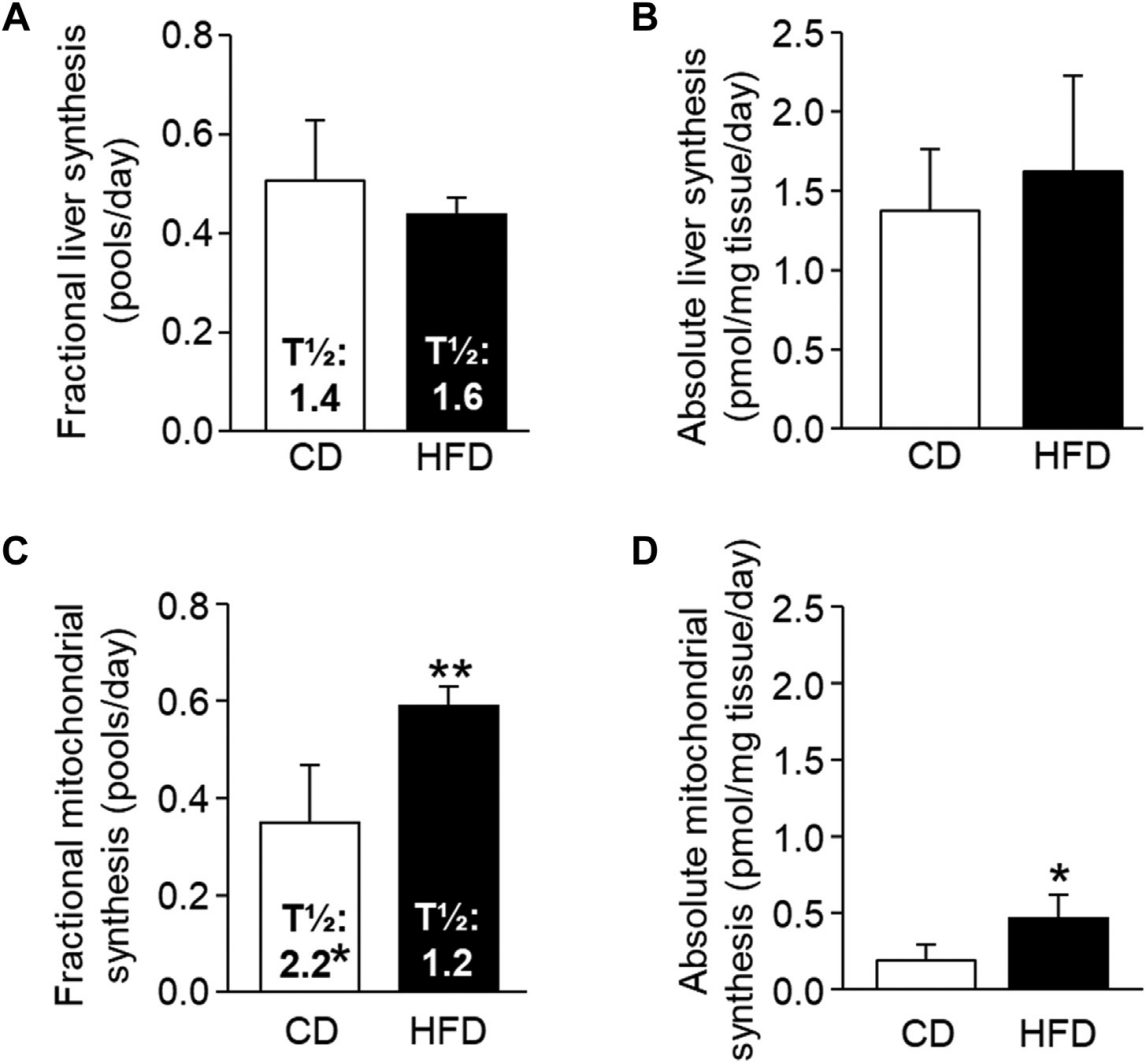
FSRs and ASRs of 16:0 CER in whole liver homogenate and isolated hepatic mitochondria. Data are calculated as FSR (pools/day) and ASR (pmol/mg tissue/day). Enrichment data (Fig. 3) were fitted to a single exponential curve to calculate FSRs. Half-lives (*t*½) were calculated as the natural log of two divided by FSR and ASRs, the product of fractional synthesis and the total 16:0 pool size for either liver or mitochondria. A and B: Whole liver homogenate 16:0 CER (A) FSRs and (B) ASRs. C and D: Isolated hepatic mitochondrial 16:0 CER (C) FSRs and (D) ASRs. Unpaired, two-tailed t-tests. CD (white bars) verses HFD (black bars): #*P* < 0.10, ∗*P* < 0.05, ∗∗*P* < 0.01, ∗∗∗*P* < 0.001.

Newly made CER within liver and hepatic mitochondria were labeled with ^13^C_3_, ^15^N l-serine, and the average percentage enrichment of labeled 16:0 CER across days within the diet groups is presented in Fig. 3. As anticipated, enrichment across labeling days significantly increased (*P* < 0.0001), with enrichments of days 2–12 being significantly higher than day 0 (unlabeled). Animals consuming the HFD tended to reach plateau enrichments earlier (∼2–4 days of labeling) than CD-fed animals (between 4 and 7 days). For turnover calculations, same-sex mice within a diet group were matched across labeling days to generate individual enrichment curves (individual curves not shown). Fractional whole liver CER 16:0 synthesis in pools per day was similar between diet groups (Fig. 4A), whereas isolated mitochondrial 16:0 fractional synthesis was significantly greater in HFD animals (Fig. 4C). In CD animals, when comparing mitochondrial to liver 16:0 synthesis in pools per day (Fig. 4, A vs. C), mitochondrial fractional synthesis was 31% *lower* than hepatic fractional synthesis rate. In HFD animals, mitochondrial fractional synthesis was 34% *higher* than hepatic fractional synthesis, demonstrating the mitochondrial pool had greater turnover than other hepatic CER 16:0 pools only in HFD animals. The half-life (*t*½) for total liver 16:0 CER was similar between diets (Fig. 4A), while mitochondrial half-life was significantly longer with CD feeding (Fig. 4C). ASRs in pmol/mg tissue/day were calculated as the product of the total 16:0 pool size and FSRs. HFD animals demonstrated similar ASRs than CD in liver (15%, Fig. 4B) and significantly greater synthesis in the mitochondrial pool (59%, Fig. 4D). Sex differences in 16:0 CER synthesis are presented in Supplemental Table S5. Reflecting total pool size, female HFD-fed mice had the greatest absolute 16:0 synthesis in both liver and mitochondrial fractions although because of the nature of the kinetic data analysis, no formal statistical analysis could be performed. Free serine enrichment was measured in total liver homogenate, and plateau enrichment was achieved within 2 days of labeling and maintained through day 12 (1.0–1.5% enrichment free hepatic serine, supplemental Fig. S5).

### Hepatic protein and mitochondrial content

**Fig. 5:**
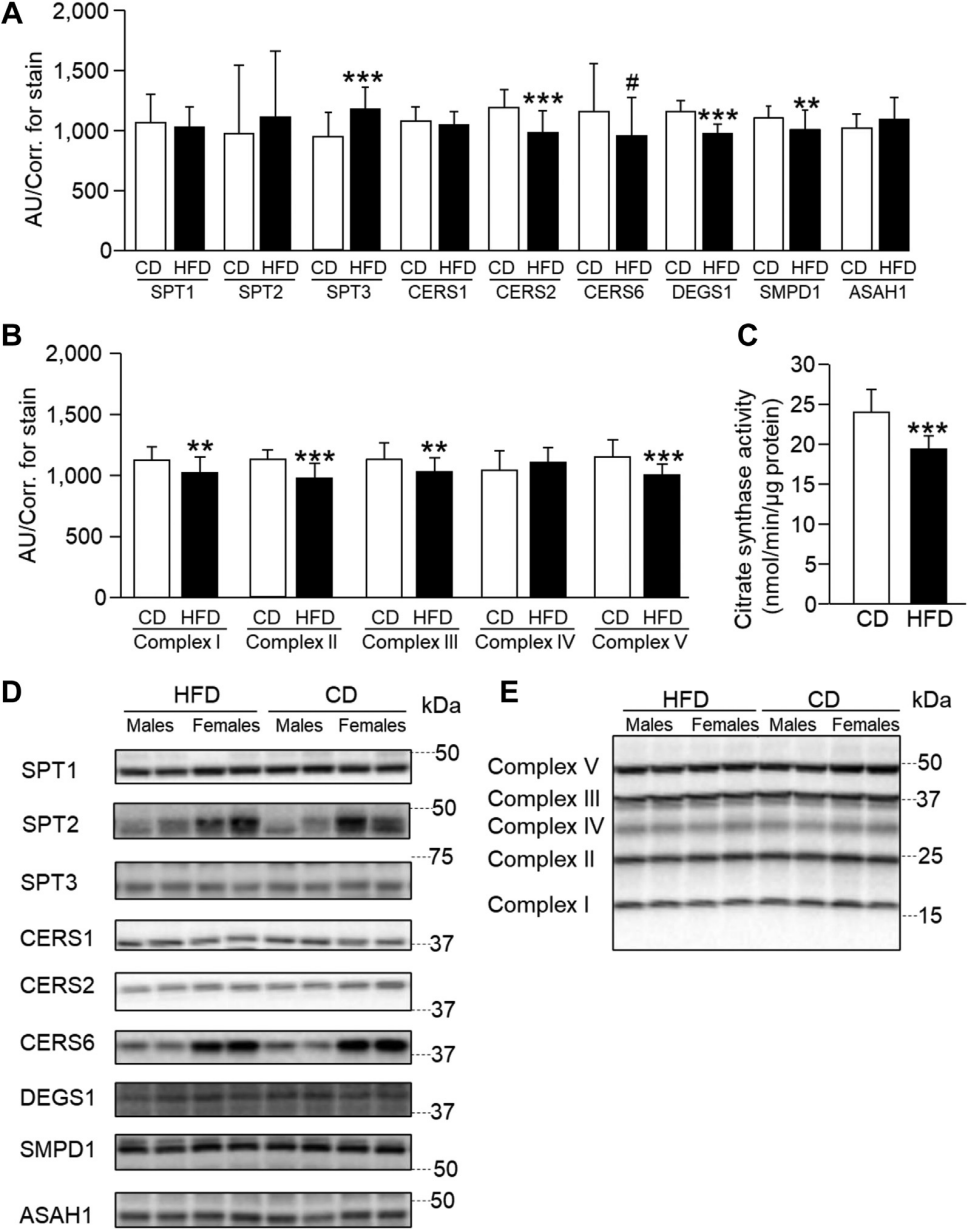
Protein expression of key CER synthetic enzymes, mitochondrial complexes, and mitochondrial citrate synthase activity in whole liver homogenate. Data are mean ± SD; *n* = 24/diet, unless otherwise noted—data from male and female mice within a diet group were averaged for each bar graph; Unpaired two-tailed *t*-tests. CD (white bars) versus HFD (black bars): #*P* < 0.10, ∗*P* < 0.05, ∗∗*P* < 0.01, ∗∗∗*P* < 0.001. A: Protein content of CER synthetic enzymes, acid sphingomyelinase (SMPD1), and acid ceramidase (ASAH1, *n* = 23/diet) in whole liver homogenate. B: Protein content of the oxidative phosphorylation complexes in whole liver homogenate. C: Citrate synthase activity in whole liver homogenate. D: Representative blots for the target CER synthetic proteins for each diet (all from whole liver homogenate). The full blots (including the subsets shown here) can be found at the end of the supplemental data section. E: Representative blots for the electron transport chain complexes (all from whole liver homogenate). The full blot (including the subset shown here) can be found at the end of the supplemental data section.

Liver protein expression of SPT, the first step in CER biosynthesis, was similar between the diet groups for subunits SPT1 and SPT2, whereas SPT3 was greater with HFD feeding (Fig. 5A). CERS1, responsible for CER 18:0 synthesis, also did not differ between HFD and CD animals, whereas CERS2 (CER 20:0+) was significantly decreased (−22%) and CERS6 (CER 14:0 and 16:0) tended to be lower (−21%) in HFD animals. Upon analysis of CERS in isolated mitochondria, no differences were found between diet groups (supplemental Fig. S6). Protein content of DEGS1 was 20% lower in HFD animals. Acid sphingomyelinase (SMPD1), a key step in the sphingomyelinase CER synthetic pathway, was lower in HFD animals (−10%), and acid ceramidase (ASAH1), an enzyme within the CER salvage pathway that converts CER to sphingosine, was not different between groups (+6%). Markers of mitochondrial content, citrate synthase activity and mitochondrial electron transport chain complexes I–III and V, were lowered by the short-term HFD (Fig. 5, B and C). Sex differences for each of these proteins are visually shown in Fig. 5D and presented in Supplemental Table S6, which demonstrated that female animals had greater SPT1, CERS2, and CERS6, whereas male mice had greater CERS1 content. Finally, purity of mitochondrial isolation was assessed and demonstrated no contamination with other cellular organelles (supplemental Fig. S6C).

## Discussion

This article demonstrates for the first time, the kinetics of liver and mitochondrial CER synthesis using an orally delivered stable isotope of l-serine in mice. Animals consuming an HFD (60% of energy) for 2 weeks exhibited greater absolute 16:0 CER synthesis rates in both liver tissue and isolated mitochondria. These observations were mirrored by increased saturated liver CERs and total and saturated mitochondrial CERs. With the currently expanding literature supporting CERs in contributing to metabolic diseases such as type 2 diabetes, nonalcoholic fatty liver disease, and cardiovascular disease (21, 23, 24, 55), deeper knowledge of in vivo CER biology is needed. While cell culture techniques for tracking CER synthesis are established (36, 37, 38, 39, 40, 41, 42, 43), the translation to whole body models can present technical and analytical challenges. This method to quantitate in vivo CER synthesis will facilitate scientific advancements in the field by determining whether gene deletion and/or overexpression have a physiological impact on CER biology. They will aid investigations of therapies to reduce CER lipotoxicity, potentially by either lowering total content, targeting synthesis of individual species, or increasing the degradation of the sphingolipid.

### Interaction between total hepatic CERs and isolated mitochondrial CERs

Both total liver and hepatic mitochondrial pools exhibited increased saturated CERs with HFD feeding, yet only within the mitochondrial fraction were total CER concentrations significantly elevated. One explanation for this finding is the short-term feeding regimen included in the current study, whereas a second explanation is that although some CERs accumulated in the mitochondria, a portion of the hepatic CERs may have been secreted from the liver into circulation. This hypothesis is supported by prior reports of increased serum CERs with high fat feeding (30, 33) and the characterization of hepatic-derived CERs contributing significantly to the circulating CER pool (13, 56). A third interpretation is derived from work by Noguchi *et al.* (57) who found that hepatic CER 16:0 was primarily synthesized from newly made 16:0 derived from de novo lipogenesis. Thus, hepatic CER concentrations may not be as greatly impacted by dietary alterations, particularly HFDs, as that of CER within other tissues like skeletal muscle. A fourth explanation could be derived from differences in protein expression of CER synthetic enzymes. However, with the exception of SPT3, which may be more important in synthesis of CERs with different sphingoid base chain lengths (e.g., myristoyl and lauryl-CoA) (58) not examined here, we found decreased hepatic protein content of enzymes within the CER signaling pathway. For example, CERS2 was lower with the HFD leading to reductions in the CER 24:0 concentrations as previously reported with longer HFD interventions (59). Whereas, in isolated mitochondria, we found no differences in the CERS between diet groups. Despite these findings, animals consuming the HFD demonstrated a robust increase in total mitochondrial CERs, and the amount of CERs within whole liver homogenate was positively correlated with the hepatic mitochondrial pool size. These observations support a dietary effect on the storage or synthesis of hepatic CERs with preference for the mitochondrial pool. Even with similar total hepatic contents, 2 weeks of a 60% fat diet elicited elevations in mitochondrial CERs. Little is known about the mechanisms responsible for cellular CER distribution within cells. Given that these CERs have a high prevalence in all cellular membranes, we anticipated that the CER quantity found in the mitochondrial pool would be only a fraction of the total liver CER pool. We document here that this fraction varied from 12% to 19%. Future studies should investigate whether CER synthetic enzymes are differentially expressed within hepatic mitochondria with longer dietary interventions, which may contribute to variation in cellular depots of CER and ultimately, if greater quantities in the mitochondrial pool are associated with cellular metabolic dysfunction.

### Turnover of hepatic and mitochondrial CER

Using this method, continuous incorporation of ^13^C_3_, ^15^N l-serine into the backbone of liver and mitochondrial 16:0 CERs was detected. CER 16:0 enrichments reached steady state in both groups around day 4. Using labeling curves generated from individual mice throughout the labeling period, exponential models were fit to the enrichment data and produced FSRs. FSRs and ASRs of liver 16:0 CER were similar between CD and HFD animals. Using labeled water, Chen *et al.* (33) reported a 5–10 fold greater plasma 16:0 CER ASR in C57Bl/6 mice fed a 45% HFD when compared with normal chow. Key differences between the current and Chen’s study include the length of the diet (12 vs. the 2 weeks used here) and the targeted pool(s) in which CER synthesis was measured (plasma vs. liver and mitochondria). Strong data from humans (60) and animals (13, 60) support the liver as the primary source of plasma CER (60–80%), which may then reflect total hepatic CER synthesis. Compared with Chen *et al.*, we observed similar fractional turnover rates in CD animals (∼0.5 vs. ∼0.6 pools/day), whereas the HFD elicited much lower fractional hepatic turnover (∼0.4 pools/day) than previously reported (∼1.1 pools/day). Results from the CD animals support future studies to compare the plasma and hepatic pools to determine their relationship.

In contrast to the liver results, synthesis of mitochondrial CER 16:0 was significantly different between the diet groups, with HFD animals having 1.6-fold greater synthetic rates than the CD animals. Mitochondrial 16:0 CER accounted for 19% to 23% of the 16:0 liver pool in both groups; thus, it is evident that the other hepatic 16:0 CER pools (e.g., endoplasmic reticulum, cellular membranes) must be turning over at different rates (some much slower) in HFD-fed animals. The reciprocal can be applied to CD animals who demonstrated 31% greater total liver fractional synthesis when compared with isolated liver mitochondria. In other words, with greater total hepatic turnover than mitochondrial-specific turnover, the other hepatic pools must have had greater synthetic rates with CD feeding. Future studies should investigate the mechanisms driving intracellular CER turnover in states of excess energy intake. Finally, the dhCER turnover data measured here objectively support dhCER as markers of CER synthetic rates, inferred by others (13). Specifically, total liver and mitochondrial dhCER 16:0 content mirrored the observed changes in 16:0 turnover; both hepatic dhCER 16:0 concentrations and fractional synthesis were unchanged between diet groups, whereas the HFD animals exhibited significantly higher mitochondrial 16:0 dhCERs as well as 16:0 fractional turnover.

### Limitations and conclusion

In addition to increasing the length of the high-fat feeding period and quantifying serum CER concentrations, future studies should pair isotopic labeling with functional assessments (e.g., respiration rates, apoptosis, reactive oxygen species production) to determine if alterations in CER synthesis are related to in vivo mitochondrial function. With respect to the method, the l-serine isotope could be used to measure synthesis of many CER species, and this method may be now optimized for alternative species although CERs in very small quantities (along with dhCERs) will require more sensitive techniques to minimize background noise. While the serine label will become incorporated into dhCER and CER species, synthesis of other complex sphingolipids like deoxyceramides can be measured with neither this method nor those with varying sphingoid base chain lengths.

In summary, strong genetic and pharmacologic evidence supports CER contribution to the development of many metabolic diseases. With the rapidly evolving knowledge of CERs, methods for tracking CER synthesis will be essential to advancements in this area. This report provides proof of principle for an approach to investigate whether early increases in mitochondrial CERs are implicated in mitochondrial dysfunction.

## Data availability

Data are available from the corresponding author upon request.

## Supplemental data

This article contains supplemental data (61, 62).

## Conflict of interest

The authors declare that they have no conflicts of interest with the contents of this article.
