# Supplementary material for: Relationship between hepatic and mitochondrial ceramides: a novel in vivo method to track ceramide synthesis
Source: J Lipid Res. 2023 Apr 5;64(5):100366. doi: 10.1016/j.jlr.2023.100366 (PMC10193228; doi:10.1016/j.jlr.2023.100366)
Supplement: Supplemental Tables S1–S6 and Figures S1–S6 [file mmc1.pdf]

SUPPLEMENTAL INFORMATION:

Relationship between hepatic and mitochondrial ceramides: Novel in vivo method to track ceramide synthesis

Justine M. Mucinski, PhD[1]; Jonas M. McCaffrey[1]; R. Scott Rector, PhD[1,2,3]; Takhar Kasumov, PhD[4,5]; Elizabeth J. Parks, PhD[1,2,*]

[1] Department of Nutrition and Exercise Physiology, University of Missouri, Columbia, MO
[2] Department of Medicine, Division of Gastroenterology and Hepatology, School of Medicine, University of Missouri, Columbia, MO
[3] Research Service, Harry S Truman Memorial Veterans Medical Center, Columbia, MO
[4] Department of Gastroenterology and Hepatology, Cleveland Clinic, Cleveland, OH
[5] Department of Pharmaceutical Sciences, College of Pharmacy, Northeast Ohio Medical University, Rootstown, OH

*CORRESPONDING AUTHOR:
Elizabeth J. Parks, PhD
One Hospital Drive School of Medicine NW 406
University of Missouri
Columbia, Missouri 65212
Email: parksej@missouri.edu

Comments on Methodology: Selection of tracer and analytical challenges

Studies measuring CER kinetics have used D_2O (33), palmitate (e.g., U-^{13}C, d_3) (42, 46-48, 61),or L-serine (e.g., U-^{13}C, d_3, $^{13}C_3$,^{15}N) (13, 33, 40, 41) isotopes to quantify synthesis, however many of these measurements were completed using cell culture models. Recently, Chen and colleagues were the first to use deuterated water (D_2O) to quantify plasma CER flux in mice (33). While the utility of D_2O supports its use for measurements of hepatic and plasma CER kinetics, multiple analytical challenges limit the use of labeled water for CER kinetic studies. The incorporation of deuterium into a whole molecule like CER produces complicated labeling patterns (i.e., the potential for M1, M2, M3, and so on) that are challenging to interpret. Additionally, no study has completed the basic cell culture experiments to identify the maximum number of deuterium labels any individual CER may gain through metabolism and synthesis (i.e., 'polymerization factor' or 'n'). Together these complications are driven by the ubiquitous use of deuterium in many metabolic processes. Regardless of these considerations, D_2O deserves further testing and optimization for the quantification of CER biosynthesis and future studies should build upon the method described by Chen et al to expand to tissue CER turnover and application in human CER synthesis. To date, the only translational studies in humans were targeted to skeletal muscle CER 16:0 synthesis using infusions of ^{13}C palmitate (46-48). In addition to human skeletal muscle, an infusion of labeled palmitate has been applied to quantify CER synthesis within skeletal muscle and liver tissue in mice (34, 35, 62). While reducing the analytical challenges associated with D_2O, for palmitate, the method delivery adds an additional technical hurdle. Infusions over extended periods of time (six+ hours) are challenging in rodent models due to the invasive nature of the procedure and the difficulty associated with repeated blood and tissue samples in the same animal.

In sum, as more data are generated implicating CERs in many metabolic conditions, methods to measure turnover are becoming increasingly crucial to better understand CER biology. The choice of stable isotope and the method of delivery deserve consideration when designing in vivo flux studies, particularly for analysis of whole molecules with complicated metabolism like CERs. The method presented here

represents the first in depth investigation into total hepatic and liver mitochondrial turnover using an oral serine stable isotope.

Protein quantification and organelle detection – antibodies

Primary antibodies used are as follows: serine palmitoyl transferase 1 (SPTLC1; Santa Cruz #374143, anti-mouse monoclonal IgG_1; 1:1,000 dilution), serine palmitoyl transferase 2 (SPTLC2; Santa Cruz #398704, anti-mouse monoclonal IgM; 1:1,000 dilution), serine palmitoyl transferase 3 (SPTLC3; AbCam #237532, anti-rabbit polyclonal; 1:1,000 dilution), dihydroceramide desaturase (DES1/FADS7; Santa Cruz #134338, anti-mouse monoclonal IgM; 1:1,000 dilution), ceramide synthase 1 (CERS1/LASS1; Sigma Aldrich SAB2104843, anti-rabbit polyclonal; 1:1,000 dilution), ceramide synthase 2 (CERS2/LASS2; Santa Cruz #390745, anti-mouse monoclonal IgG_1; 1:1,000 dilution), ceramide synthase 6 (CERS6/LASS6; Santa Cruz #100554, anti-mouse monoclonal IgG_{2a}; 1:1,000 dilution), acid sphingomyelinase (SMPD1; Bio-Rad #AHP3001, anti-rabbit polyclonal IgG; 1:1,000 dilution), and acid ceramidase (ASAH1; Sigma-Aldrich #ABN468, anti-rabbit polyclonal; 1:1,000 dilution). The oxidative phosphorylation complexes I-V were also quantified by western blot (Total OxPhos cocktail; AbCam #ab110413, anti-mouse; 1:1,000 dilution). Secondary antibodies used include HRP-linked anti-mouse (#7076S, Cell Signaling, Danvers, MA) and anti-rabbit (#7074S, Cell Signaling, Danvers, MA) IgG at 1:5,000 dilution. The organelle detection cocktail (Organelle Detection Western Blot Cocktail; AbCam #133989; 1:250 dilution) was used to confirm the purity of the mitochondrial isolation with appreciable binding occurring only with ATP5A, the mitochondrial marker (**supplementary figure 6**).

Statistical analysis – R code

```
## Example orthogonal contrasts as used in figure 3 (used where appropriate, e.g., type III tests)
Data$day<-as.factor(Data$day)
d0vd1= c(-1, 1, 0, 0, 0, 0)
d0vd2= c(-1, 0, 1, 0, 0, 0)
d0vd4= c(-1, 0, 0, 1, 0, 0)
d0vd7= c(-1, 0, 0, 0, 1, 0)
d0vd12= c(-1, 0, 0, 0, 0, 1)
contrasts(Data$day) = cbind(d0vd1, d0vd2, d0vd4, d0vd7, d0vd12)
Data$day


## ANOVA
library(stats)
library(car)
AOV_model1 = aov(y ~ Time*diet, data = Data)
Anova(AOV_model1, type = "III")

# Post hoc pairwise t-test with Bonferroni correction (as in figure 3)
AOV_model1_PH<-Data %>%  pairwise_t_test(y ~ x, paired = TRUE, p.adjust.method = "bonferroni")
AOV_model1_PH

## Correlation
library(Hmisc)
rcorr(x, y, type=c("pearson"))

##T-Test
library(stats)
t.test(x, y, alternative="two.sided")

##Assumptions
#normality
shapiro.test(residuals(model1))
#Homogeneity of Variance
library(car)
leveneTest(Data$y, Data$Time)
leveneTest(Data$y, Data$sex)
leveneTest(Data$y, Data$diet)
leveneTest(Data$y, interaction(Data$Time, Data$sex, Data$diet))
```

Supplementary table 1. LC/MS transitions and retention times for all CER and dhCER species analyzed

**Ceramide**	**Transition (*m/z*)**	**Retention time (minutes)**
18:1/17:0	552.6 → 264	4.42
18:1/14:0	510.5 → 264	4.10
18:1/16:0	538.5 → 264	4.32
18:1/18:0	566.4 → 264	4.50
18:1/18:1	564.4 → 264	4.35
18:1/20:0	594.4 → 264	4.69
18:1/22:0	622.6 → 264	4.84
18:1/24:0	650.6 → 264	4.98
18:1/24:1	648.6 → 264	4.81
**Labeled Ceramide**	**Transition (*m/z*)**	**Retention time (minutes)**
18:1/16:0- M1	539.5 → 265	4.32
18:1/16:0- M3	541.5 → 267	4.32
**Dihydroceramide**	**Transition (*m/z*)**	**Retention time (minutes)**
18:0/16:0	540.6 → 266	4.40
18:0/18:0	568.4 → 266	4.57
18:0/24:0	652.6 → 266	4.98
18:0/24:1	650.6 → 266	4.88

Considerations for calculating the fraction of the total liver or mitochondrial CER pool made new over the 12-day experiment

Supplementary figure 1. Collision induced ionization of CER 16:0

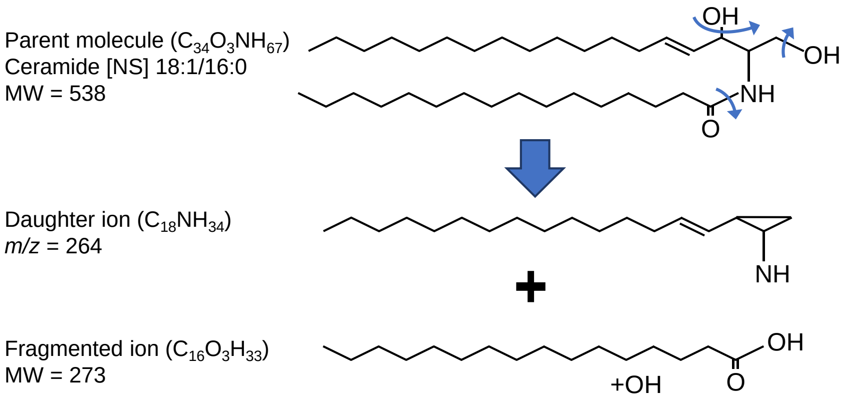

Three isotopomers of the daughter ion (M0, M1, M3), shown in **supplementary figure 1** were monitored. Note that no standard curve of enrichment is available for this molecule, thus we considered a number of factors in monitoring newly-made CER using the serine backbone. **Supplementary table 2** presents the proportions of the isotopomers of the CER fragment due to natural abundance. The proportions were normalized such that their sum equaled 100%. It is assumed a pool that is 100% new that has been made with $^{13}C_3$, ^{15}N serine, would have a m/z of 267, resulting from one lost ^{13}C during the condensation of serine and palmitate. The excess M4, M5, etc. (due to other naturally occurring isotopes) were not monitored.

Supplementary table 2. Proportions of the isotopomers of the CER fragment

Natural abundance of daughter ion		
M0 (m/z 264)	M1 (m/z 265)	M3 (m/z 267)
82.988%	16.929%	0.083%
Fully-labeled daughter ion		
M0 (m/z 264)	M1 (m/z 265)	M3 (m/z 267)
0%	0%	100%

The proportion of the pool made new was calculated two ways.
Calculation 1: For each sample, the observed raw area ratios of M3 / (sum M0, M1, M3) was calculated. This ratio can overestimate the fraction of the pool made new due to the small amount of background enrichment in M3 present in nature (0.083%).

Calculation 2: This calculation used M1% and M3%. As shown in **supplementary table 3** below, the proportion of the pool made new was modeled based on mixtures of cold (pool 1) and labeled (pool 2) pools. The natural abundance and fully-labeled pools were mixed at increments of 1% from 0% new to 20% new. Next, a relationship was calculated between the proportion of the pool that is new and the ratio of M3/M1 in the mixture.

Supplementary table 3. Mixture of pool 1 (natural abundance) and pool 2 (fully-labeled)

**Normalized natural abundance**				**Fully-labeled**				**Mixtures of pools 1 and 2**						
Pool 1				*Pool 2*										
**M0**	**M1**	**M3**		**M0**	**M1**	**M3**		Cold	hot CER	**M0**	**M1**	**M3**		
264	265	267		264	265	267								**Ratio of M3/M1**
82.988%	16.929%	0.083%		0.000%	0.000%	100.000%		100.0%	0.0%	82.99%	16.93%	0.08%		0.005
82.988%	16.929%	0.083%		0.000%	0.000%	100.000%		99.0%	1.0%	82.16%	16.76%	1.08%		0.065
82.988%	16.929%	0.083%		0.000%	0.000%	100.000%		98.0%	2.0%	81.33%	16.59%	2.08%		0.125
82.988%	16.929%	0.083%		0.000%	0.000%	100.000%		97.0%	3.0%	80.50%	16.42%	3.08%		0.188
82.988%	16.929%	0.083%		0.000%	0.000%	100.000%		96.0%	4.0%	79.67%	16.25%	4.08%		0.251
82.988%	16.929%	0.083%		0.000%	0.000%	100.000%		95.0%	5.0%	78.84%	16.08%	5.08%		0.316
82.988%	16.929%	0.083%		0.000%	0.000%	100.000%		94.0%	6.0%	78.01%	15.91%	6.08%		0.382
82.988%	16.929%	0.083%		0.000%	0.000%	100.000%		93.0%	7.0%	77.18%	15.74%	7.08%		0.450
82.988%	16.929%	0.083%		0.000%	0.000%	100.000%		92.0%	8.0%	76.35%	15.58%	8.08%		0.519
82.988%	16.929%	0.083%		0.000%	0.000%	100.000%		91.0%	9.0%	75.52%	15.41%	9.08%		0.589
82.988%	16.929%	0.083%		0.000%	0.000%	100.000%		90.0%	10.0%	74.69%	15.24%	10.07%		0.661
82.988%	16.929%	0.083%		0.000%	0.000%	100.000%		89.0%	11.0%	73.86%	15.07%	11.07%		0.735
82.988%	16.929%	0.083%		0.000%	0.000%	100.000%		88.0%	12.0%	73.03%	14.90%	12.07%		0.810
82.988%	16.929%	0.083%		0.000%	0.000%	100.000%		87.0%	13.0%	72.20%	14.73%	13.07%		0.888
82.988%	16.929%	0.083%		0.000%	0.000%	100.000%		86.0%	14.0%	71.37%	14.56%	14.07%		0.966
82.988%	16.929%	0.083%		0.000%	0.000%	100.000%		85.0%	15.0%	70.54%	14.39%	15.07%		1.047
82.988%	16.929%	0.083%		0.000%	0.000%	100.000%		84.0%	16.0%	69.71%	14.22%	16.07%		1.130
82.988%	16.929%	0.083%		0.000%	0.000%	100.000%		83.0%	17.0%	68.88%	14.05%	17.07%		1.215
82.988%	16.929%	0.083%		0.000%	0.000%	100.000%		82.0%	18.0%	68.05%	13.88%	18.07%		1.302
82.988%	16.929%	0.083%		0.000%	0.000%	100.000%		81.0%	19.0%	67.22%	13.71%	19.07%		1.390
82.988%	16.929%	0.083%		0.000%	0.000%	100.000%		80.0%	20.0%	66.39%	13.54%	20.07%		1.482

Mixing pool 1 and pool 2, an exponential formula was generated to predict a rising M3%/M1% as the amount of newly-labeled pool 2 was increased (**supplementary figure 2**). We then put the observed M3%/M1% from each sample into the formula to predict the proportion of the pool made new during the experiment.

Supplementary figure 2. Predicted M3%/M1% vs actual newly-made fraction

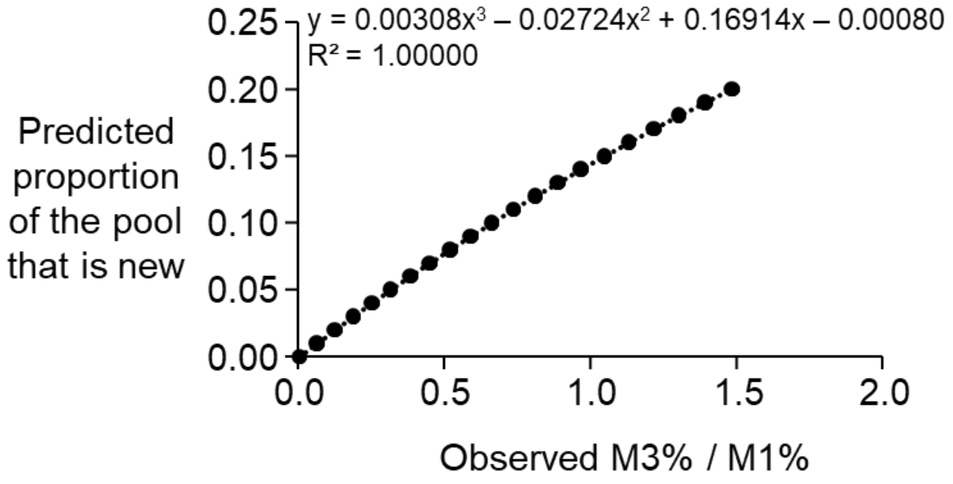

As expected, we found good agreement between the raw enrichments observed (**calc. 1**, above) and the M3/M1 ratio (**calc. 2**, above). The observed %E from day-12 animals was measured to be 1.9 ± 0.1%, while the model using the M3/M1 ratio predicted that 2.0 ± 0.1% would have been made new. Thus, adding the M3/M1 ratio did not improve the calculation substantially. Thus, future studies may simply monitor the fraction of M3% / sum of M0 + M3 and subtract natural abundance of 0.083%.

Supplementary table 4. Sex differences in whole liver homogenate and isolated hepatic mitochondrial ceramide concentrations

pmol/mg tissue	Control Diet		High Fat Diet	
**Liver CERs**	Male	Female	Male	Female
18:1/14:0 #	0.50 ± 0.02	0.54 ± 0.04	0.49 ± 0.04	0.52 ± 0.04
18:1/16:0 *,#	2.01 ± 0.42	3.61 ± 0.77	2.70 ± 0.60	4.60 ± 1.37
18:1/18:0 *,#,§	0.93 ± 0.11	2.50 ± 0.77	1.68 ± 0.51	5.16 ± 2.04
18:1/18:1	0.77 ± 0.03	0.77 ± 0.04	0.75 ± 0.05	0.76 ± 0.04
18:1/20:0 *,§	3.99 ± 2.48	5.34 ± 2.26	11.66 ± 4.46	8.42 ± 3.94
18:1/22:0#	152.70 ± 15.23	146.32 ± 5.45	155.16 ± 16.45	146.22 ± 9.74
18:1/24:0	136.72 ± 76.36	145.21 ± 35.09	109.65 ± 26.20	138.21 ± 43.31
18:1/24:1 #	194.33 ± 85.55	261.15 ± 60.56	203.78 ± 51.63	270.53 ± 94.13
Total#	491.95 ± 175.56	565.43 ± 89.29	485.88 ± 92.19	574.41 ± 139.22
**Liver dhCERs**				
18:0/16:0 #	1.02 ± 0.12	1.30 ± 0.41	0.96 ± 0.15	1.30 ± 0.33
18:0/18:0 *,#	0.11 ± 0.07	0.32 ± 0.19	0.17 ± 0.09	0.44 ± 0.17
18:0/24:0	57.92 ± 64.77	62.30 ± 23.67	30.65 ± 13.61	50.83 ± 19.84
18:0/24:1#	114.92 ± 73.07	183.59 ± 83.18	105.39 ± 33.70	203.70 ± 74.07
Total	173.98 ± 133.93	247.51 ± 84.69	137.17 ± 44.52	256.28 ± 78.50
**Mitochondrial CERs**				
18:1/14:0 #	0.03 ± 0.01	0.04 ± 0.01	0.03 ± 0.01	0.04 ± 0.01
18:1/16:0 *,#	0.35 ± 0.07	0.71 ± 0.13	0.58 ± 0.08	0.98 ± 0.20
18:1/18:0 *,#,§	0.14 ± 0.04	0.63 ± 0.31	0.40 ± 0.10	1.37 ± 0.38
18:1/18:1 *,#	0.04 ± 0.01	0.05 ± 0.01	0.05 ± 0.01	0.06 ± 0.01
18:1/20:0 *,#,§	0.93 ± 0.41	1.24 ± 0.69	4.90 ± 1.45	2.81 ± 1.13
18:1/22:0 *,#,§	10.37 ± 1.83	11.72 ± 1.93	21.78 ± 3.97	15.39 ± 1.96
18:1/24:0 *,#	14.73 ± 7.38	21.06 ± 7.29	33.64 ± 7.78	43.84 ± 16.59
18:1/24:1 #	21.08 ± 6.62	40.26 ± 15.71	29.12 ± 18.99	41.97 ± 26.33
Total *,#	47.69 ± 15.07	75.72 ± 22.89	90.50 ± 28.84	106.47 ± 45.34
**Mitochondrial dhCERs**				
18:0/16:0 *,#	0.08 ± 0.01	0.14 ± 0.02	0.13 ± 0.04	0.21 ± 0.08
18:0/18:0 *,#,§	0.01 ± 0.01	0.06 ± 0.03	0.04 ± 0.02	0.13 ± 0.03
18:0/24:0 *,#	8.09 ± 4.24	14.01 ± 4.29	22.34 ± 4.03	34.57 ± 10.50
18:0/24:1 #	13.51 ± 5.12	30.36 ± 9.90	18.73 ± 6.53	31.66 ± 11.22
Total *,#	21.70 ± 7.98	44.56 ± 12.28	41.24 ± 6.43	66.58 ± 18.59

Data are presented as mean ± SD; (*n* = 12/diet/sex) in units of pmol/mg liver tissue wet weight. ANOVA was used to explore the impacts of sex and diet on whole liver homogenate CER and dhCER; data were not corrected for multiple comparisons.
* Main effect of diet ($P < 0.05$)
# Main effect of sex ($P < 0.05$)
§ Interaction effect (P < 0.05

Supplementary table 5. CER 16:0 whole liver homogenate and isolated hepatic mitochondrial synthesis presented by sex and diet

	Control Diet		High Fat Diet	
**Liver**	Male	Female	Male	Female
Predicted asymptote (%)	2.8 ± 0.5%	2.7 ± 0.5%	2.4 ± 0.1%	2.4 ± 0.1%
FSR (*k*, pools/day)	0.57 ± 0.11	0.44 ± 0.13	0.41 ± 0.01	0.46 ± 0.01
ASR (pmol/mg tissue/day)	1.2 ± 0.4	1.6 ± 0.4	1.1 ± 0.1	2.1 ± 0.1
T½ (days)	1.2 ± 0.2	1.6 ± 0.5	1.7 ± 0.0	1.5 ± 0.0
Newly-made CER 16:0 (pmol/mg tissue)	0.051 ± 0.011	0.081 ± 0.019	0.059 ± 0.012	0.108 ± 0.022
**Mitochondrial CERs**				
Predicted asymptote (%)	2.6 ± 0.1%	2.4 ± 0.1%	3.0 ± 0.2%	3.0 ± 0.2%
FSR (*k*, pools/day)	0.34 ± 0.15	0.36 ± 0.14	0.57 ± 0.05	0.61 ± 0.02
ASR (pmol/mg tissue/day)	0.1 ± 0.1	0.3 ± 0.1	0.3 ± 0.1	0.6 ± 0.0
T½(day)	2.3 ± 1.0	2.1 ± 0.8	1.2 ± 0.1	1.1 ± 0.0
Newly-made CER 16:0 (pmol/mg tissue)	0.008 ± 0.001	0.014 ± 0.002	0.018 ± 0.003	0.030 ± 0.009

Data were calculated as fractional (pools/day) and absolute (pmol/mg tissue/day) synthetic rates (n = 12/diet/sex). Enrichment data were fitted to a single exponential curve to calculate fractional synthesis rates (FSR). Half-lives (T½) were calculated as the natural log of two divided by FSA and absolute synthetic rates are the product of fractional synthesis and the total 16:0 pool size for either liver or mitochondria. Newly-made CER 16:0 represent the average CER 16:0 made in animals at plateau enrichment – i.e., animals receiving 4-12 days of label. Due to the nature of the experiment and the average of animals within a sex and study day used to create a single curve, a single turnover rate was calculated within a diet and thus no statistical analysis could be performed for the kinetic measures.

Supplementary table 6. Sex differences for protein expression of key enzymes related to CER synthesis, oxidative phosphorylation complexes, and mitochondrial citrate synthase activity in whole liver homogenate

	**Control Diet**		**High Fat Diet**	
	Male	Female	Male	Female
Whole Liver Homogenate				
CERS1 [#]	1,143 ± 102	1,019 ± 94	1,105 ± 129	981 ± 69
CERS2 [*,#]	1,098 ± 128	1,273 ± 114	908 ± 181	1,037 ± 166
CERS6 [,#]	807 ± 155	1,500 ± 230	659 ± 102	1,249 ± 122
SPT1 [#]	978 ± 134	1,156 ± 283	908 ± 132	1,141 ± 129
SPT2 [#]	672 ± 258	1,273 ± 656	822 ± 266	1,415 ± 612
SPT3 *	950 ± 190	948 ± 218	1,183 ± 198	1,169 ± 172
DEGS1 *	1,142 ± 86	1,169 ± 95	937 ± 102	995 ± 56
SMPD1 *	1,082 ± 71	1,138 ± 111	919 ± 171	1,093 ± 105
ASAH1	1,006 ± 129	1,040 ± 92	1,047 ± 213	1,136 ± 146
Complex I *	1,135 ± 96	1,113 ± 131	1,019 ± 126	1,028 ± 139
Complex II [*,#]	1,130 ± 76	1,174 ± 80	947 ± 139	1,050 ± 80
Complex III *	1,153 ± 113	1,099 ± 154	1,024 ± 142	1,022 ± 95
Complex IV	1,031 ± 134	1,050 ± 179	1,102 ± 121	1,099 ± 130
Complex V *	1,137 ± 153	1,167 ± 142	977 ± 98	1,031 ± 71
Citrate synthase [1]	22.4 ± 2.9	25.4 ± 1.9	19.6 ± 1.5	19.0 ± 1.9
Isolated Liver Mitochondria				
CERS1	1,114 ± 391	950 ± 371	1,062 ± 628	1,026 ± 701
CERS2 [#]	593 ± 376	1,521 ± 680	886 ± 352	1,313 ± 527
CERS6 [#, §]	1,425 ± 405	1,154 ± 482	343 ± 215	2,015 ± 1090

Data are presented as arbitrary units, corrected for stain ± SD unless otherwise stated; (*n* = 12/diet/sex). Two-way, between-factors ANOVA for total liver CER and dhCER (sex and diet); supplemental data were not corrected for multiple comparisons.

[1] nmol/ minute/ mg protein

* Main effect of diet ($P < 0.05$)

Main effect of sex ($P < 0.05$)

§ Interaction effect ($P < 0.05$)

Supplementary figure 3. Proportion of isolated hepatic mitochondrial ceramides within whole liver homogenate ceramides

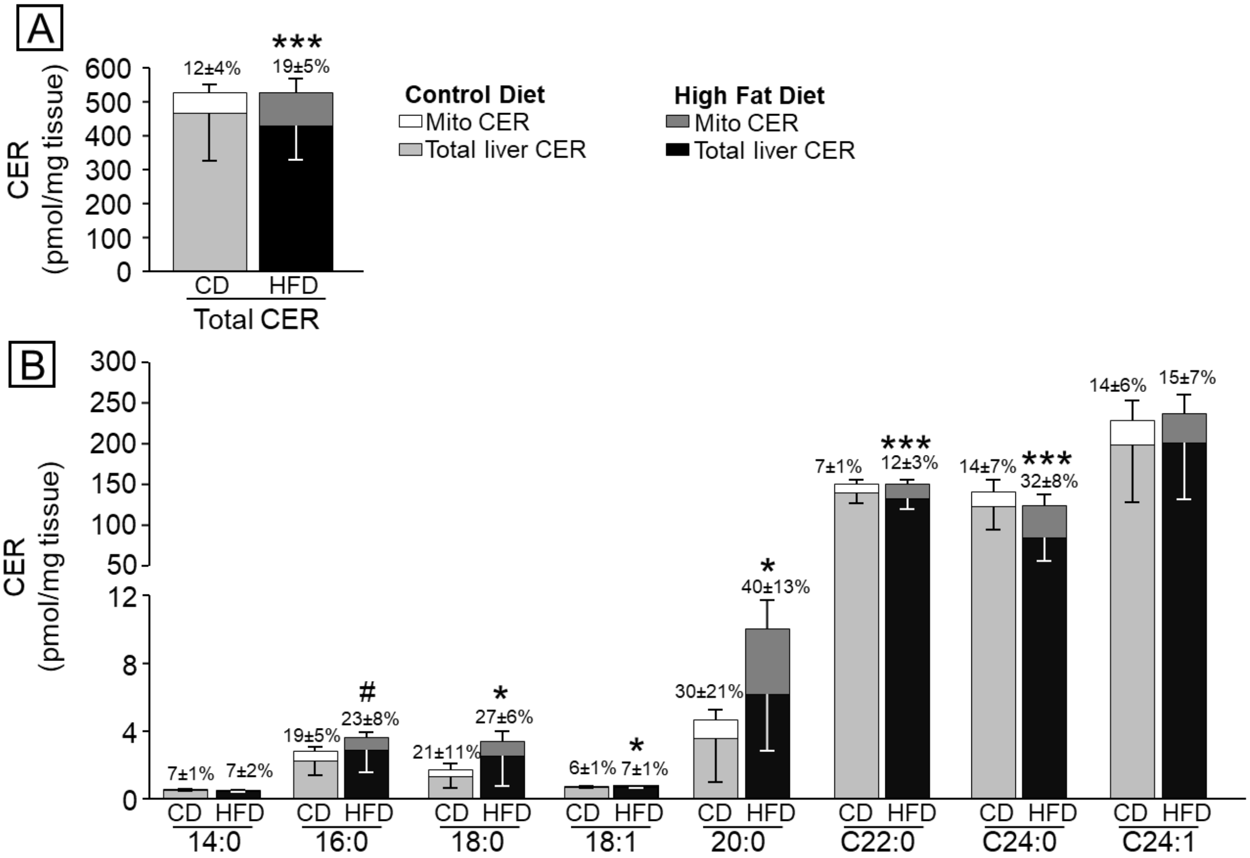

Data are presented as mean ± SD of both concentration and proportion (%) of mitochondrial CER within total liver CER; *n* = 24/diet. Unpaired, two-tailed *t*-tests. CD (isolated hepatic mitochondria: white bars & non-mito liver pools: light grey) verses HFD (isolated hepatic mitochondria: dark grey bars & non-mito liver pools: black bars) within total or individual species: # $P < 0.10$, * $P < 0.05$, ** $P < 0.01$, *** $P < 0.001$. All CER presented contain an 18:1 backbone.

A: Total hepatic CER within liver separated by mitochondrial CER and non-mitochondrial CER.

B: Individual hepatic CER species separated by mitochondrial CER and non-mitochondrial CER.

Supplementary figure 4. Whole liver homogenate and isolated hepatic mitochondrial dihydroceramide concentrations

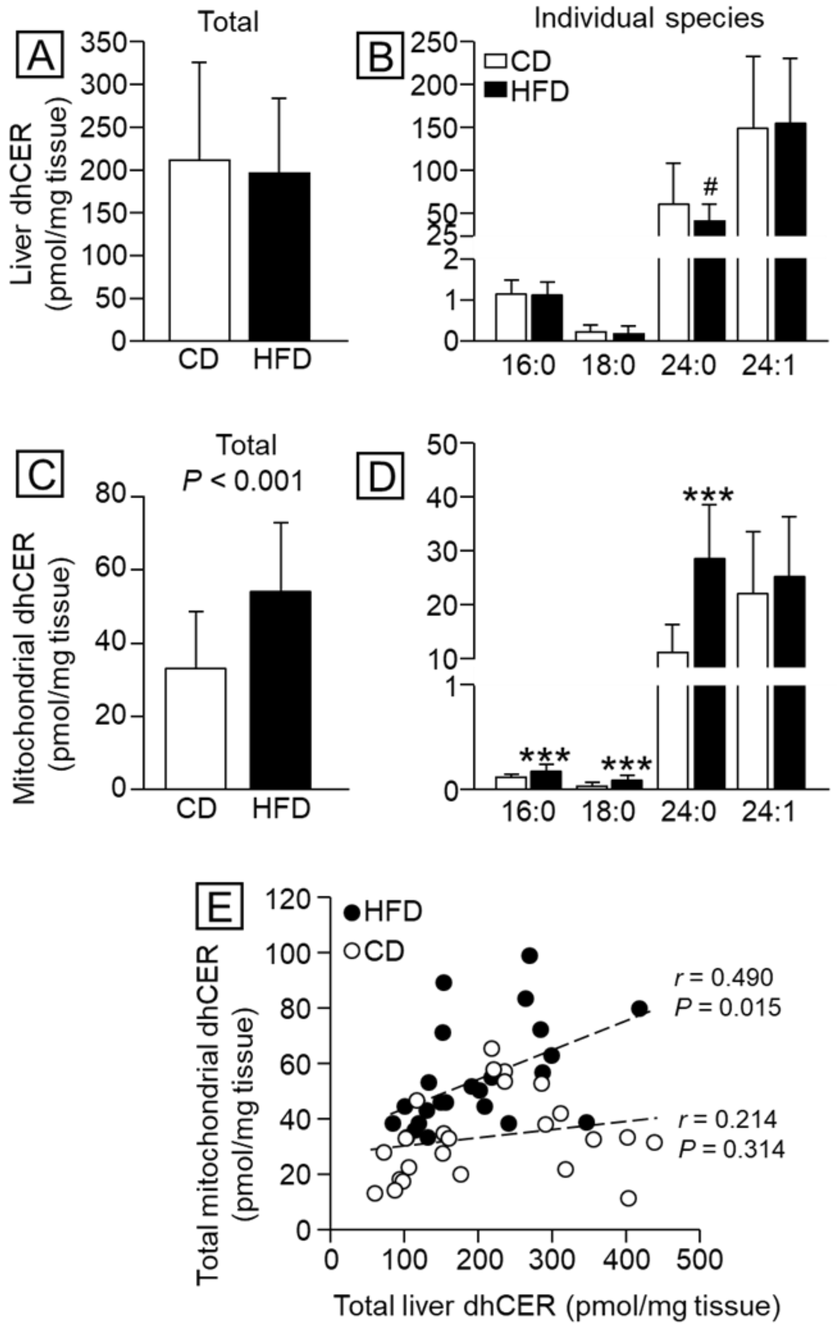

Data are presented as mean ± SD; n = 24/diet. Unpaired, two-tailed t-tests. CD (white bars) verses HFD (black bars) within total or individual species: # $P < 0.10$, * $P < 0.05$, ** $P < 0.01$, *** $P < 0.001$. All CER presented contain an 18:1 backbone.

A-B: Total or individual whole liver homogenate dhCER species.

C-D: Total or individual isolated hepatic mitochondrial dhCER species.

E: Relationship between total whole liver homogenate and isolated hepatic mitochondrial dhCER (Pearson correlation).

Supplementary figure 5. Free serine enrichment within whole liver homogenate

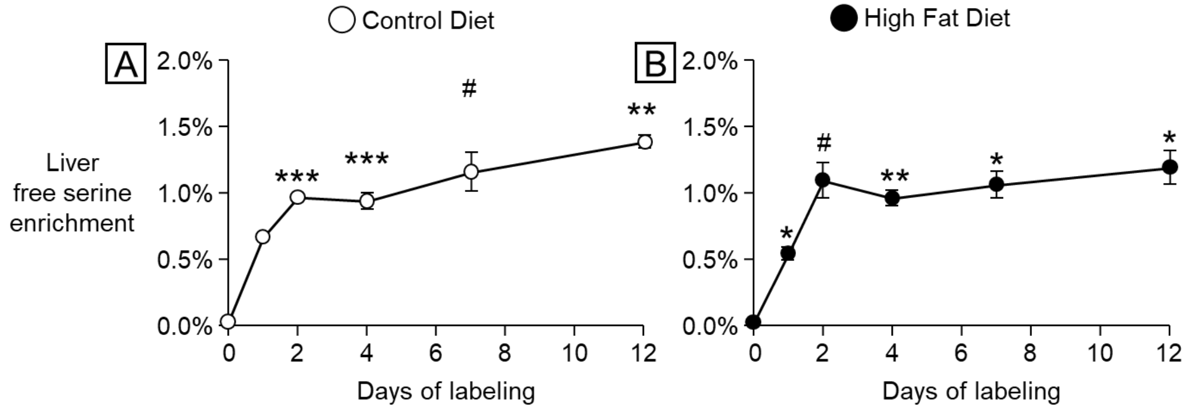

Data are presented as mean ± SEM in open circles for CD and filled circles for HFD (*n* = 2-4 mice/timepoint). ANOVA for differences across labeling days (all $P < 0.0001$) and post-hoc *t*-test with Bonferroni adjustments # $P < 0.10$, * $P < 0.05$, ** $P < 0.01$, *** $P < 0.001$ versus unlabeled (day 0).
A-B: Whole liver homogenate free serine percent enrichment across labeling days (0, 1, 2, 4, 7, 12) for CD and HFD.

Supplementary figure 6. Protein expression of key CER synthetic enzymes within mitochondria and organelle detection for presence of mitochondria within isolate

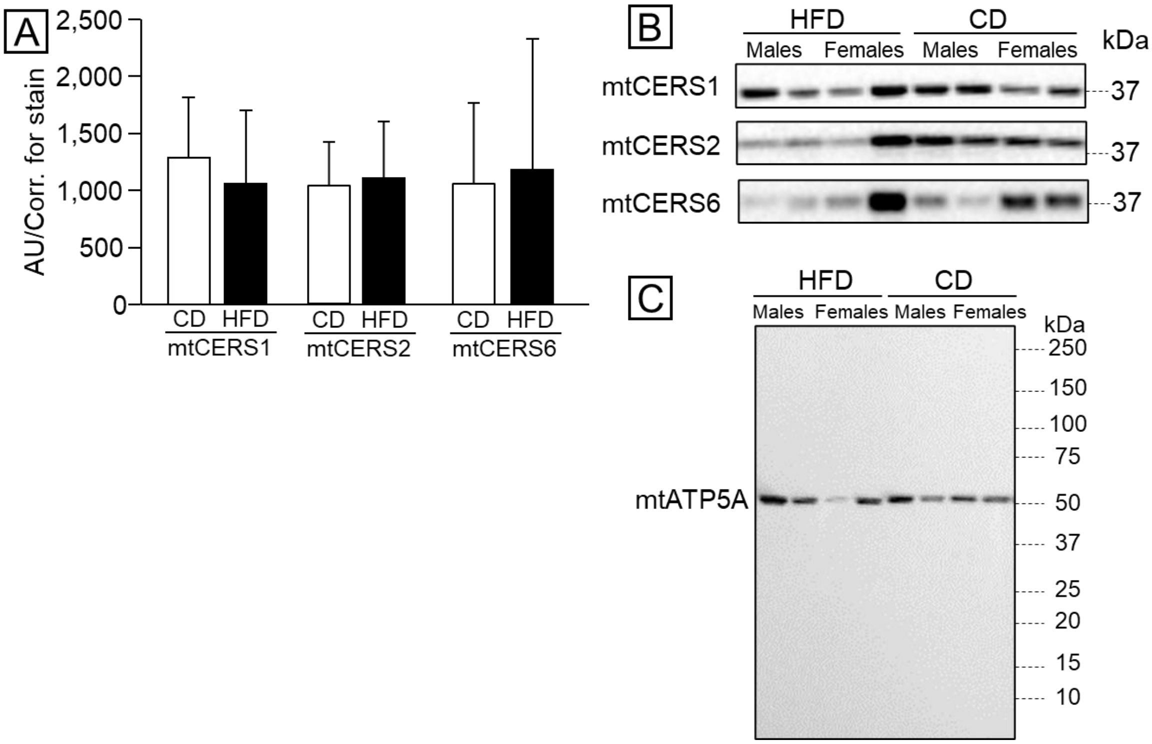

Data are mean ± SD; n = 24/diet, unless otherwise noted - data from male and female mice within a diet group were averaged for each bar graph; Unpaired, two-tailed t-tests. CD (white bars) verses HFD (black bars): # $P < 0.10$, * $P < 0.05$, ** $P < 0.01$, *** $P < 0.001$

A: Protein content of selected CER synthases in isolated liver mitochondria.

B: Representative blots for the target CER synthetic proteins (all from isolated liver mitochondria). The full blots (including the subsets shown here) can be found at the end of the supplemental section.

C: Representative blot for organelle detection. The full blot (including the subset shown here) can be found at the end of the supplemental section.

Protein quantification in whole liver homogenate – Original western blots:

Blot layouts:

4–20% Criterion™ TGX™ Precast Midi Protein Gel, 26 well, 15 µl #5671095

Blot #1 (days 0, 4, 12):

1	2	3	4	5	6	7	8	9	10	11	12	13	14	15	16	17	18	19	20	21	22	23	24	25	26
Kaleidoscope (10uL)	HFD: Male Day 0	HFD: Male Day 0	HFD: Female Day 0	HFD: Female Day 0	HFD: Male Day 4	HFD: Male Day 4	HFD: Female Day 4	HFD: Female Day 4	HFD: Male Day 12	HFD: Male Day 12	HFD: Female Day 12	HFD: Female Day 12	CD: Male Day 0	CD: Male Day 0	CD: Female Day 0	CD: Female Day 0	CD: Male Day 4	CD: Male Day 4	CD: Female Day 4	CD: Female Day 4	CD: Male Day 12	CD: Male Day 12	CD: Female Day 12	CD: Female Day 12	Kaleidoscope (5uL)

Blot #2 (days 1, 2, 7):

1	2	3	4	5	6	7	8	9	10	11	12	13	14	15	16	17	18	19	20	21	22	23	24	25	26
Kaleidoscope (10uL)	HFD: Male Day 1	HFD: Male Day 1	HFD: Female Day 1	HFD: Female Day 1	HFD: Male Day 2	HFD: Male Day 2	HFD: Female Day 2	HFD: Female Day 2	HFD: Male Day 7	HFD: Male Day 7	HFD: Female Day 7	HFD: Female Day 7	CD: Male Day 1	CD: Male Day 1	CD: Female Day 1	CD: Female Day 1	CD: Male Day 2	CD: Male Day 2	CD: Female Day 2	CD: Female Day 2	CD: Male Day 7	CD: Male Day 7	CD: Female Day 7	CD: Female Day 7	Kaleidoscope (5uL)

SPTLC1 – Whole liver Homogenate

Primary antibody: Serine palmitoyl transferase, anti-mouse monoclonal IgG_1, Santa Cruz #374143; 1:1,000 dilution
Secondary antibody: HRP-linked anti-mouse IgG, Cell Signaling #7076S, 1:5,000 dilution

Blot #1 (days 0, 4, 12):

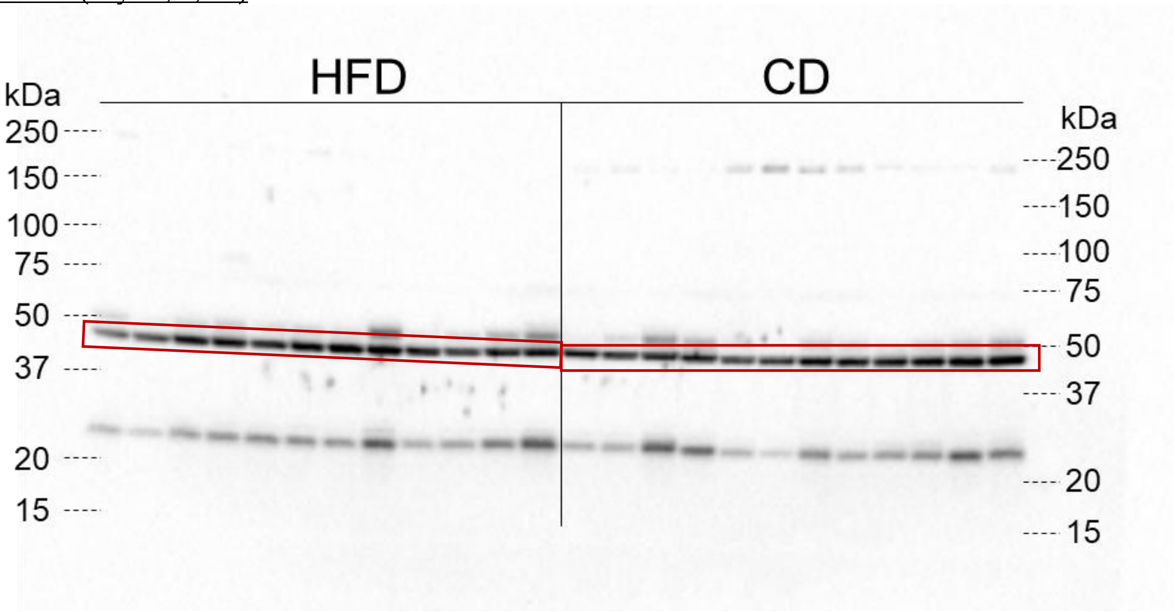

Blot #2 (days 1, 2, 7):

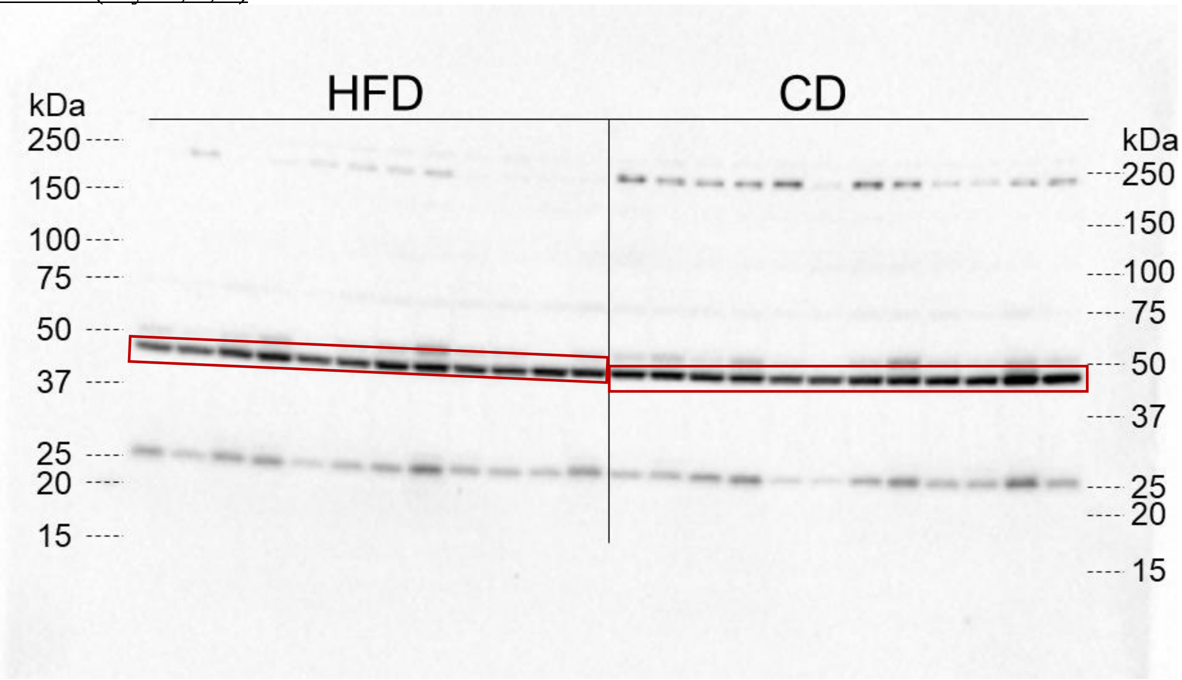

Note: A subset of this image is presented in figure 5D for representative purposes.

SPTLC2 – Whole liver Homogenate

Primary antibody: Serine palmitoyl transferase 2, anti-mouse monoclonal IgM, Santa Cruz #398704; 1:1,000 dilution
Secondary antibody: HRP-linked anti-mouse IgG, Cell Signaling #7076S, 1:5,000 dilution

Blot #1 (day 0, 4, 12):

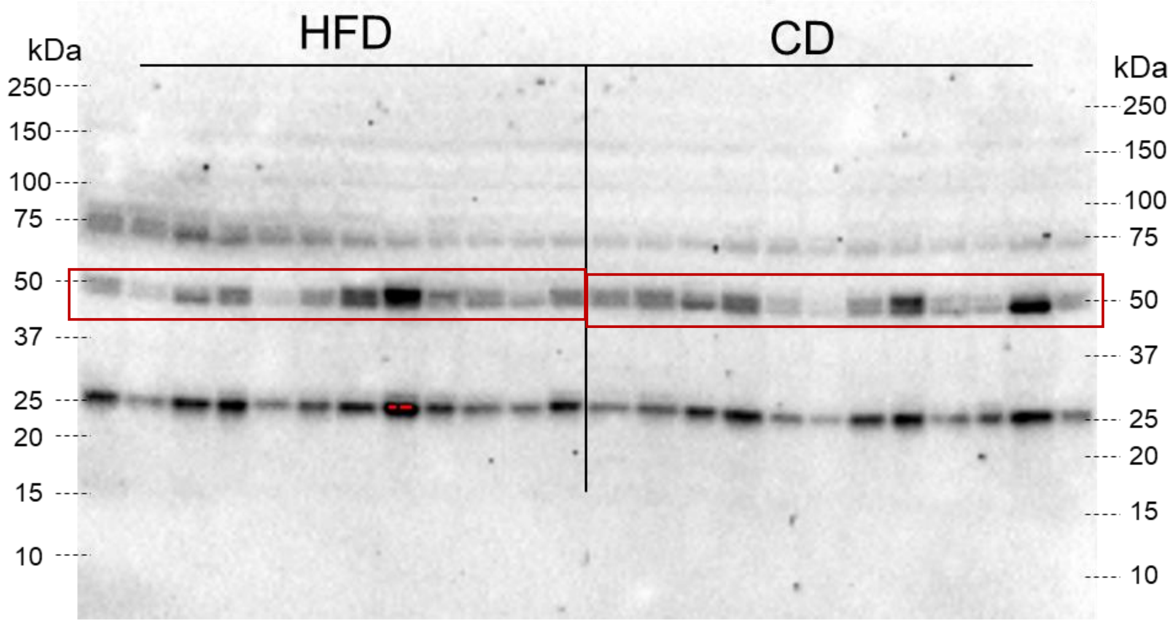

Blot #2 (day 1, 2, 7):

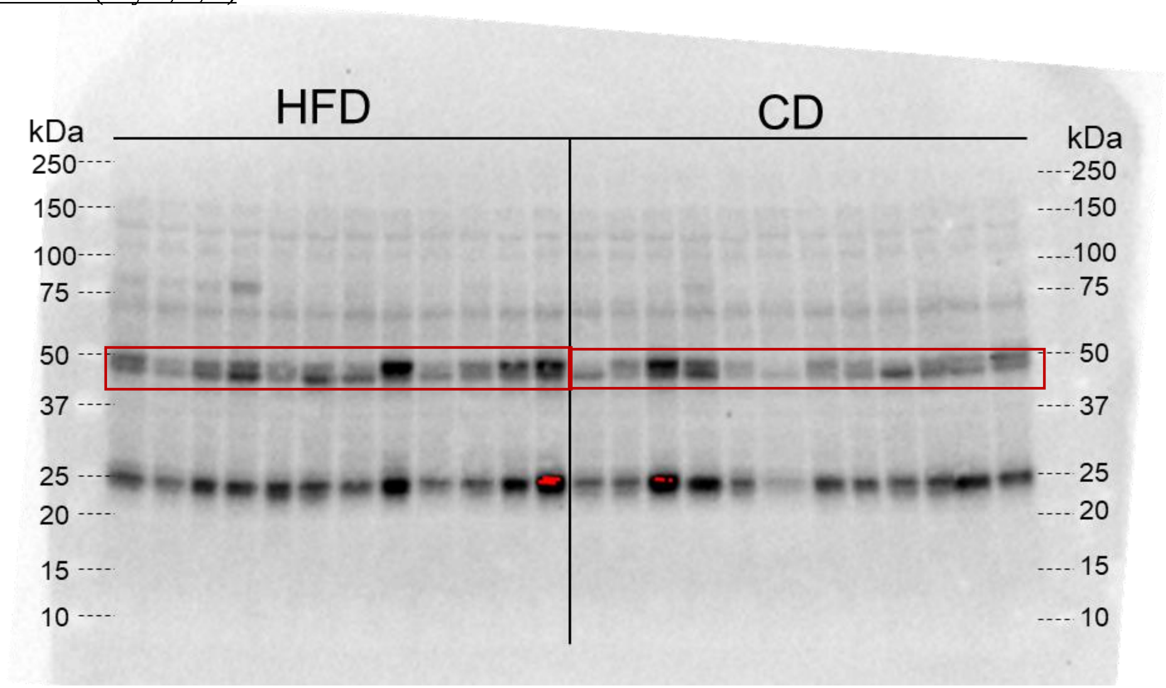

Note: A subset of this image is presented in figure 5D for representative purposes.

SPTLC3 – Whole liver Homogenate

Primary antibody: Serine palmitoyl transferase 3, anti-rabbit polyclonal, AbCam #237532; 1:1,000 dilution

Secondary antibody: HRP-linked anti-rabbit IgG, Cell Signaling #7074S, 1:5,000 dilution

Blot #1 (day 0, 4, 12):

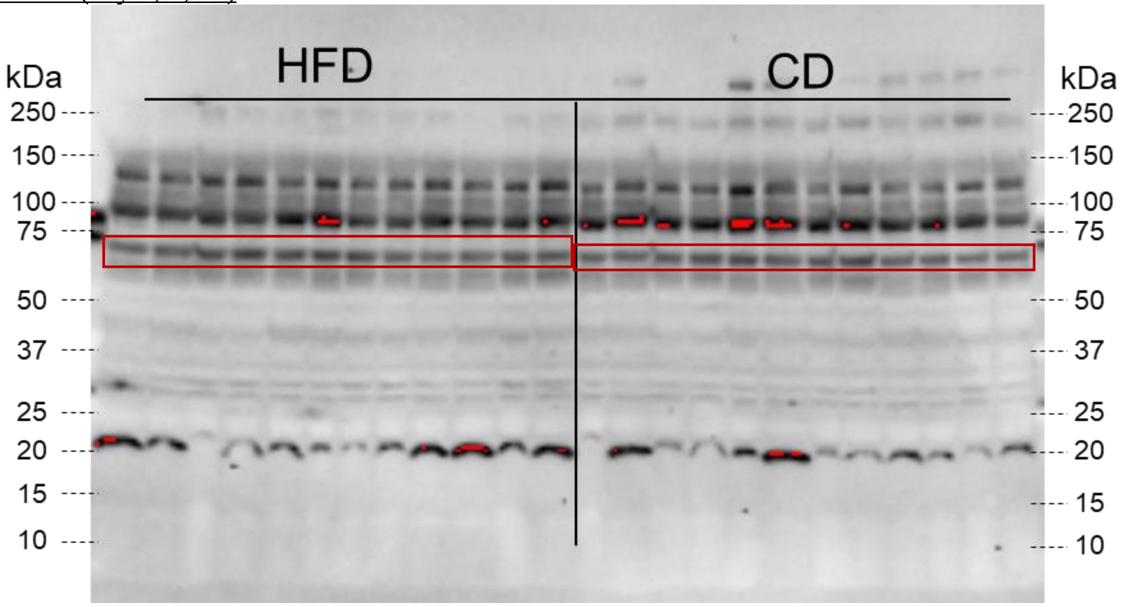

Blot #2 (day 1, 2, 7):

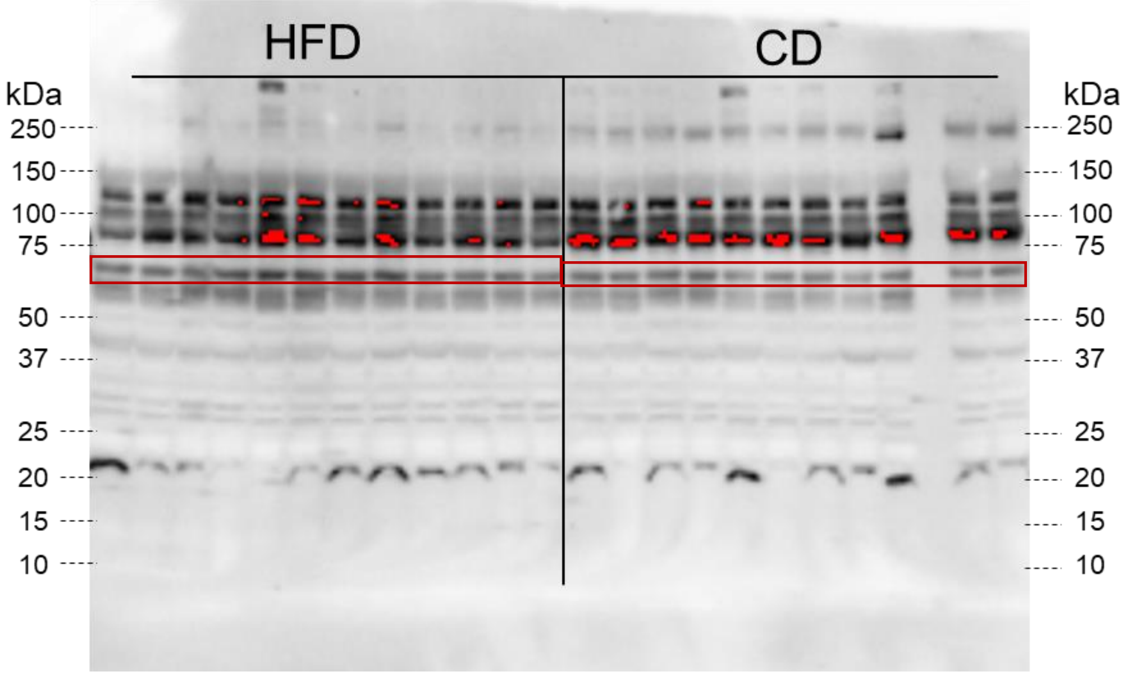

Note: No sample for Lane 22. A subset of this image is presented in figure 5D for representative purposes.

CERS1 – Whole liver Homogenate

Primary antibody: Ceramide synthase 1 (CERS1/LASS1), anti-rabbit polyclonal, Sigma Aldrich SAB2104843; 1:1,000 dilution
Secondary antibody: HRP-linked anti-rabbit IgG, Cell Signaling #7074S, 1:5,000 dilution

Blot #1 (days 0, 4, 12):

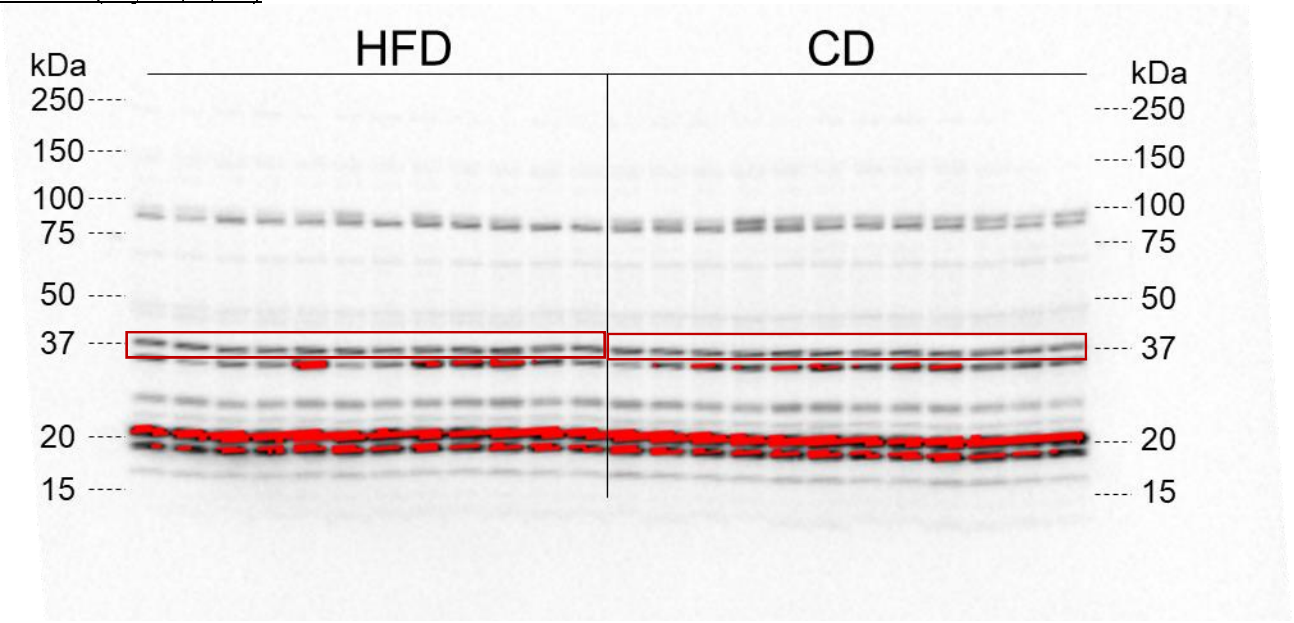

Blot #2 (days 1, 2, 7):

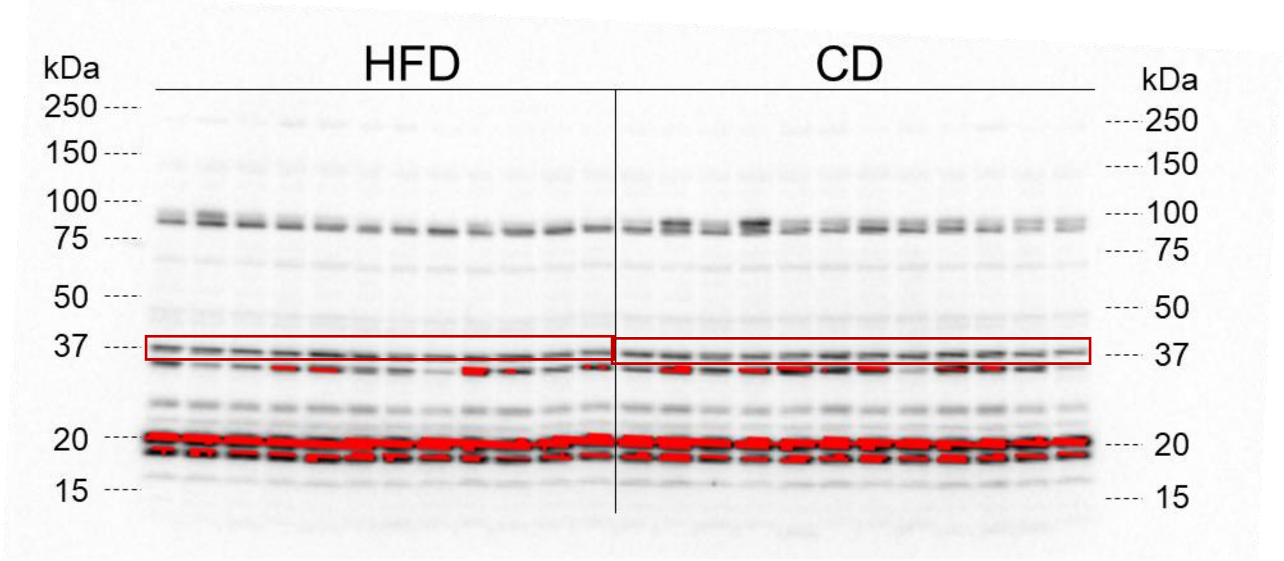

Note: A subset of this image is presented in figure 5D for representative purposes.

CERS2 – Whole liver Homogenate

Primary antibody: Ceramide synthase 2 (CERS2/LASS2), anti-mouse monoclonal IgG_1, Santa Cruz #390745; 1:1,000 dilution
Secondary antibody: HRP-linked anti-mouse IgG, Cell Signaling #7076S, 1:5,000 dilution

Blot #1 (days 0, 4, 12):

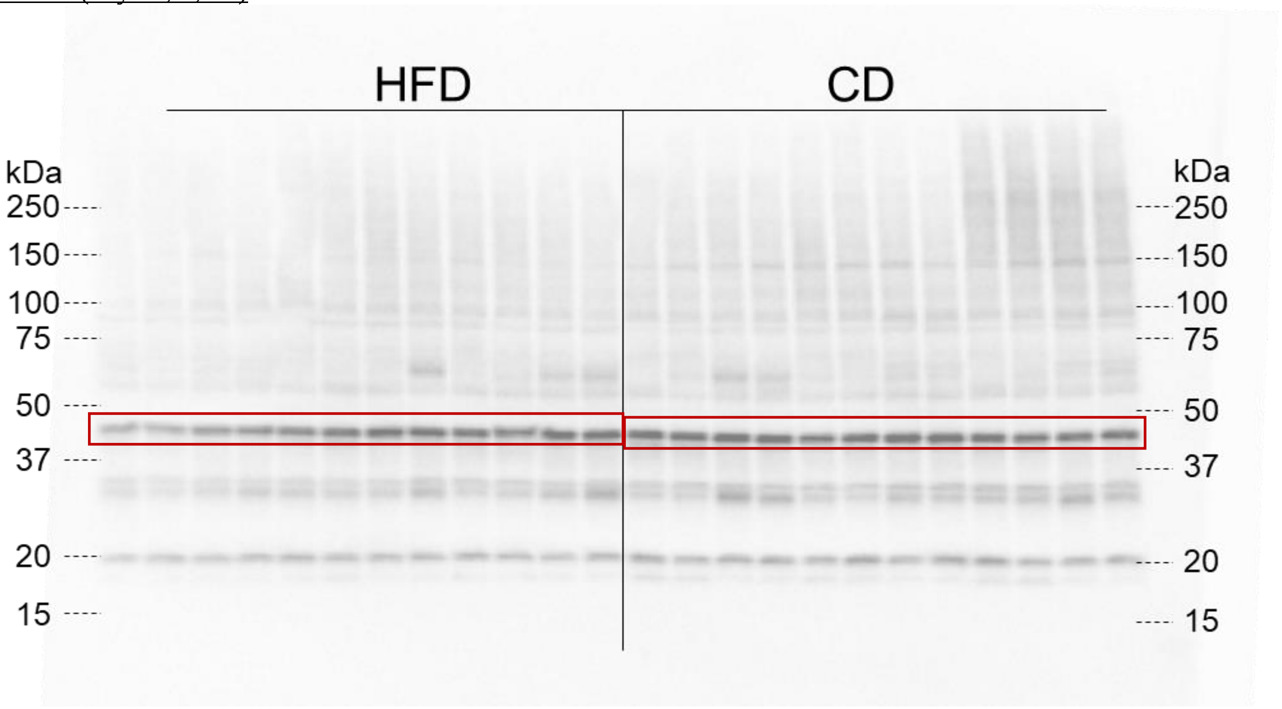

Blot #2 (days 1, 2, 7):

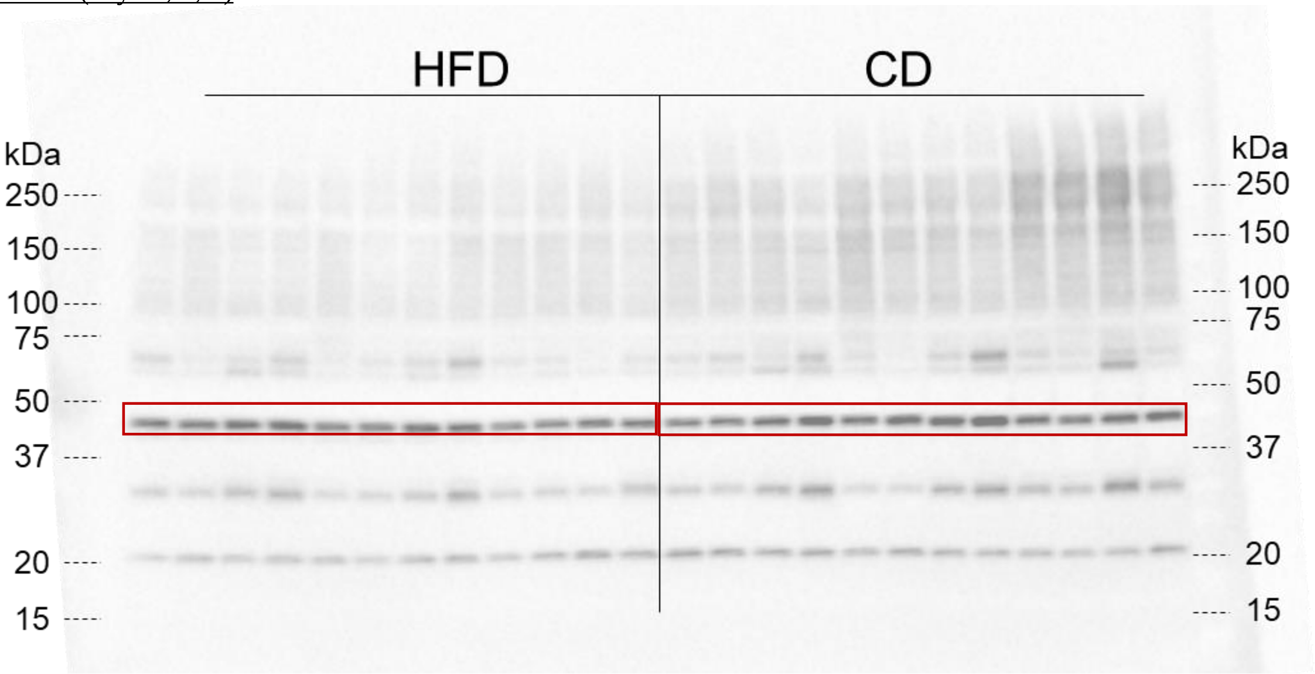

Note: A subset of this image is presented in figure 5D for representative purposes.

CERS6 – Whole liver Homogenate

Primary antibody: Ceramide synthase 6 (CERS6/LASS6), anti-mouse monoclonal IgG_{2a}, Santa Cruz #100554; 1:1,000 dilution
Secondary antibody: HRP-linked anti-mouse IgG, Cell Signaling #7076S, 1:5,000 dilution

Blot #1 (days 0, 4, 12):

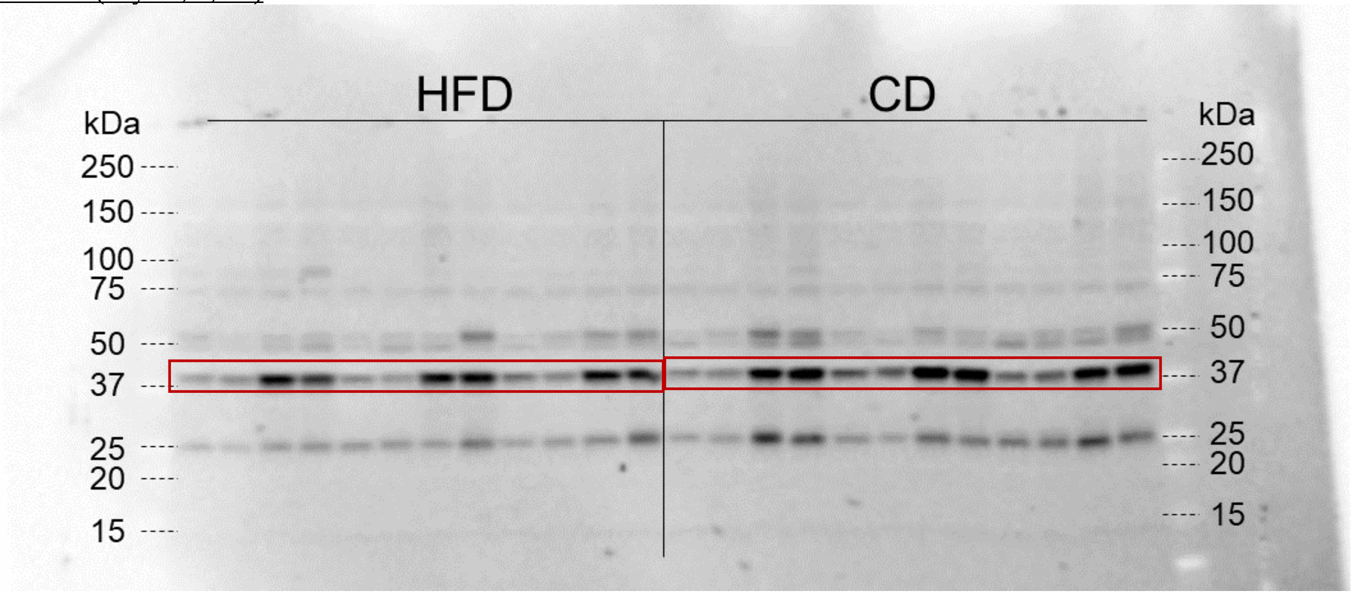

Blot #2 (days 1, 2, 7):

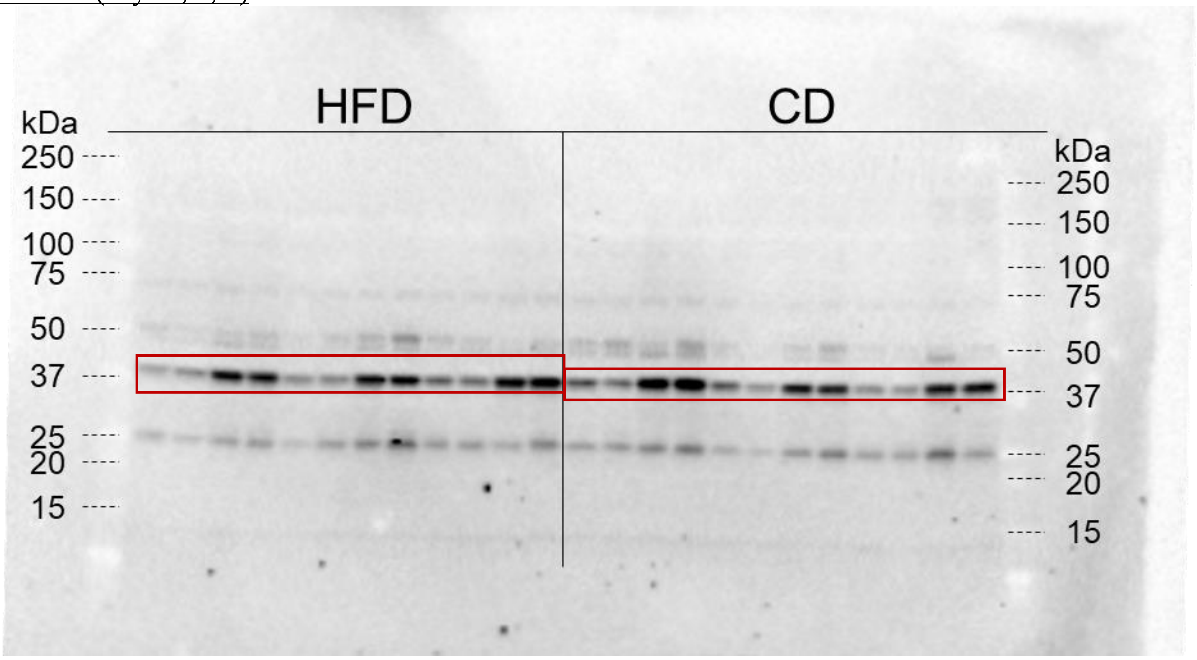

Note: A subset of this image is presented in figure 5D for representative purposes.

DEGS1 – Whole liver Homogenate

Primary antibody: Dihydroceramide desaturase (DES1/FADS7), anti-mouse monoclonal IgM, Santa Cruz #134338; 1:1,000 dilution
Secondary antibody: HRP-linked anti-mouse IgG, Cell Signaling #7076S, 1:5,000 dilution

Blot #1 (days 0, 4, 12):

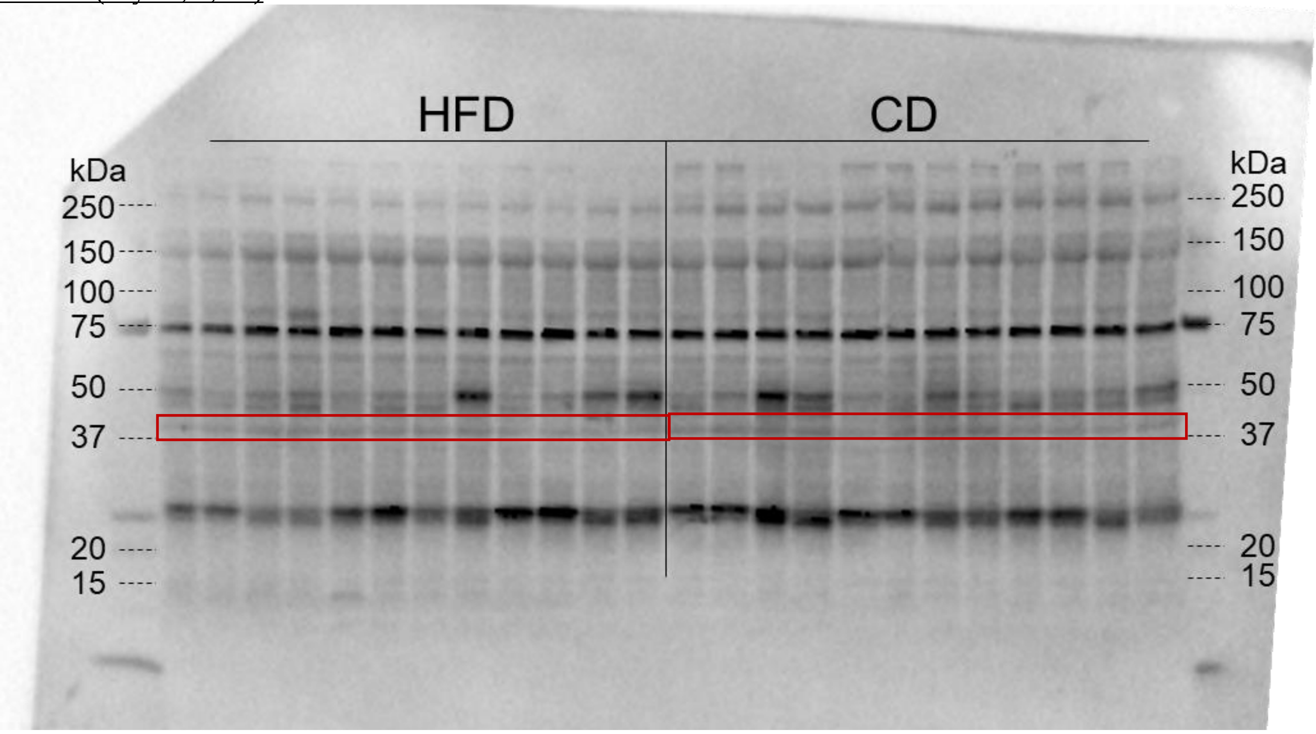

Blot #2 (days 1, 2, 7):

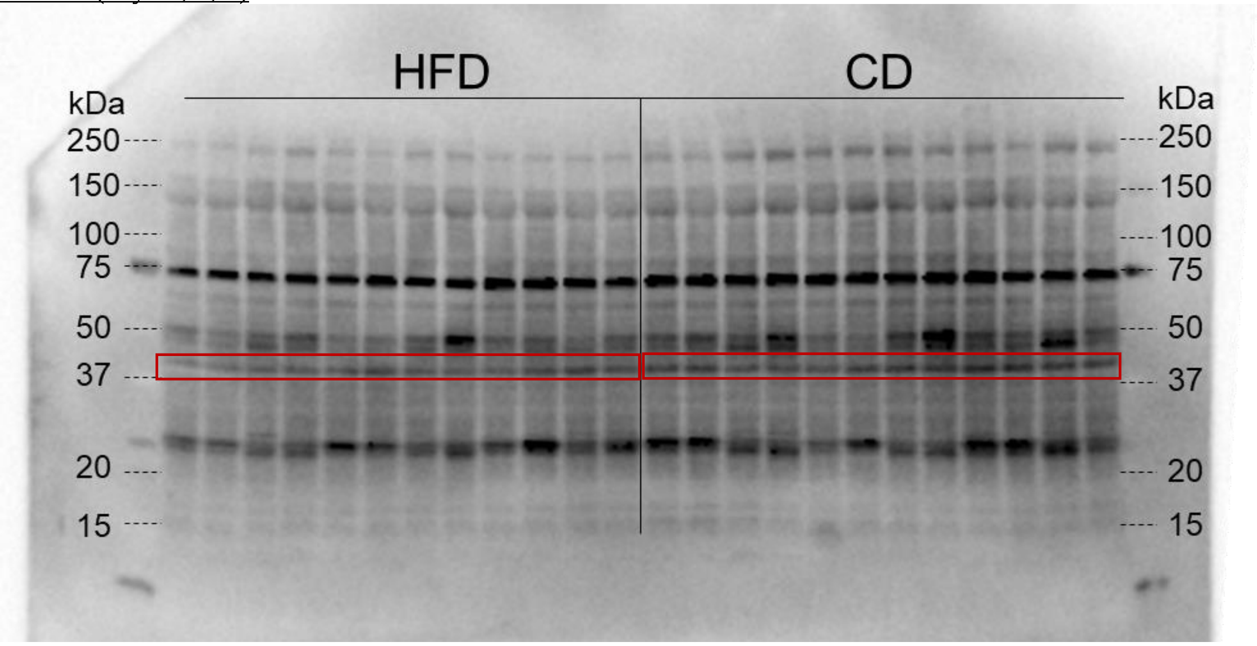

Note: A subset of this image is presented in figure 5D for representative purposes.

SMPD1 – Whole liver Homogenate

Primary antibody: Acid sphingomyelinase (SMPD1), anti-rabbit polyclonal IgG, Bio-Rad #AHP3001; 1:1,000 dilution
Secondary antibody: HRP-linked anti-rabbit IgG, Cell Signaling #7074S, 1:5,000 dilution

Blot #1 (days 0, 4, 12):

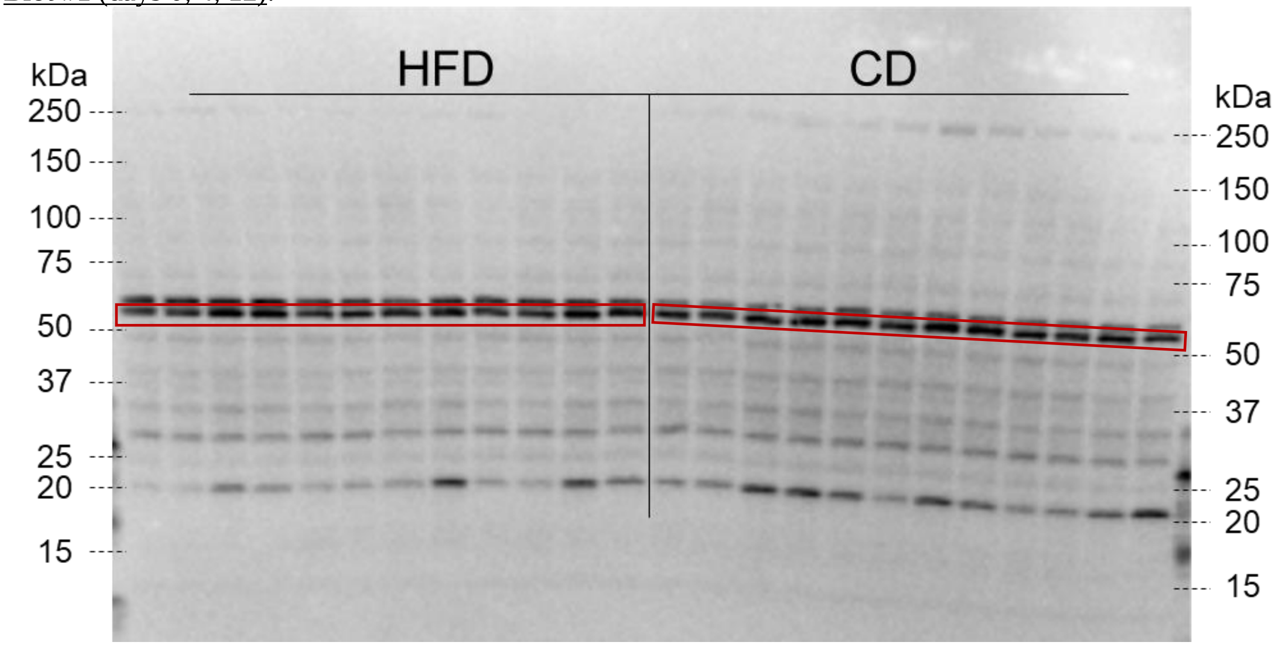

Blot #2 (days 1, 2, 7):

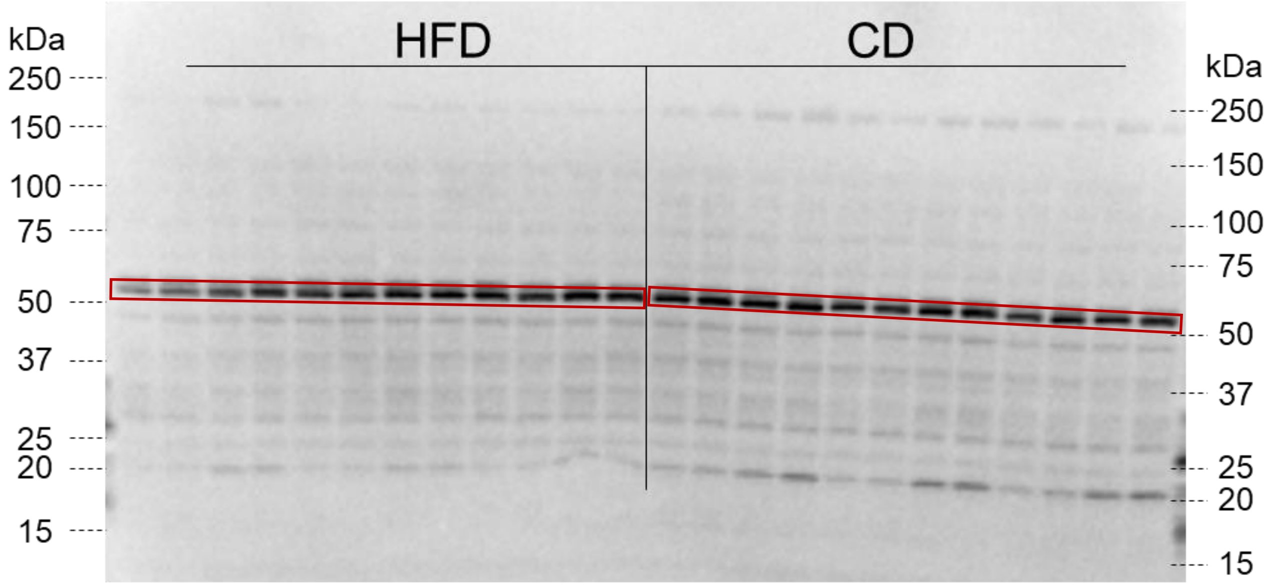

Note: A subset of this image is presented in figure 5D for representative purposes.

ASAH1 – Whole liver Homogenate

Primary antibody: Acid ceramidase (ASAH1), anti-rabbit polyclonal, Sigma-Aldrich #ABN468; 1:1,000 dilution
Secondary antibody: HRP-linked anti-rabbit IgG, Cell Signaling #7074S, 1:5,000 dilution

Blot #1 (days 0, 4, 12):

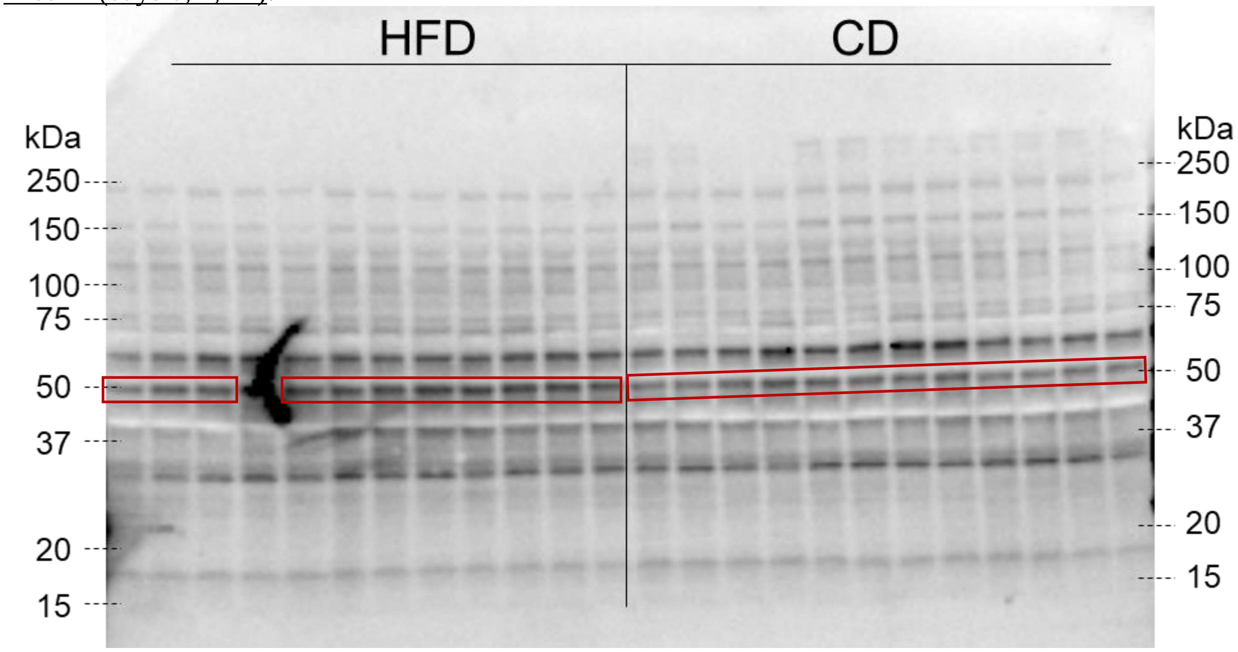

Note: Lane #5 was not quantified due to blot interference.

Blot #2 (days 1, 2, 7):

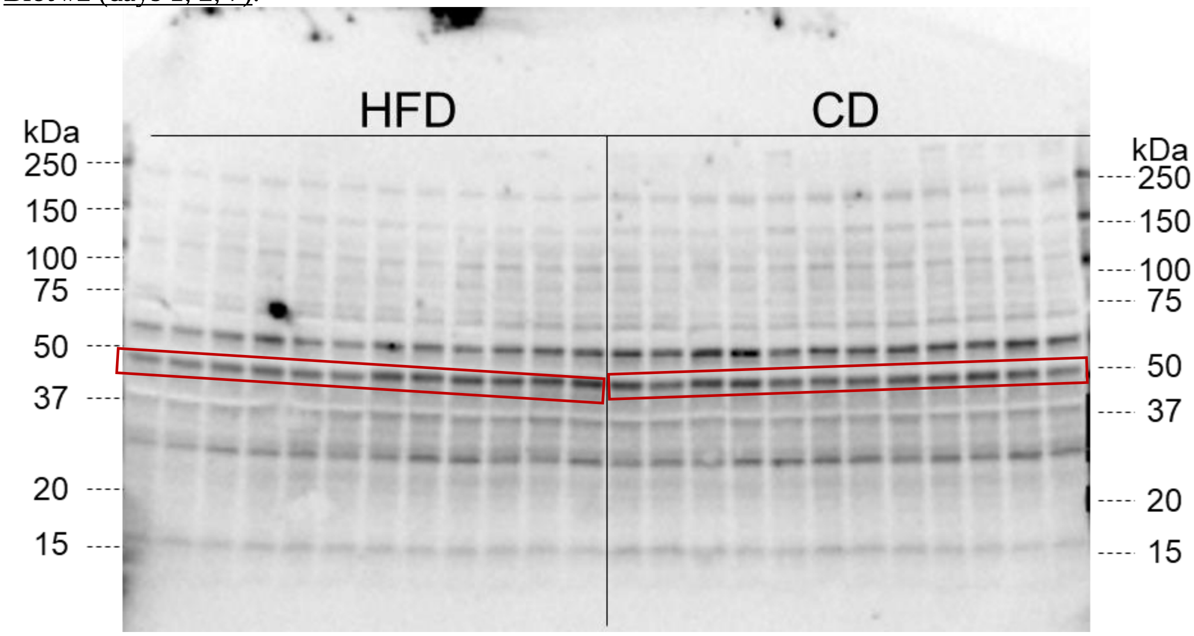

Note: A subset of this image is presented in figure 5D for representative purposes.

OXPHOS Complexes I-V – Whole liver Homogenate

Primary antibody: Total OxPhos cocktail, anti-mouse, AbCam #ab110413; 1:1,000 dilution
Secondary antibody: HRP-linked anti-mouse IgG, Cell Signaling #7076S, 1:5,000 dilution

Blot #1 (days 0, 4, 12):

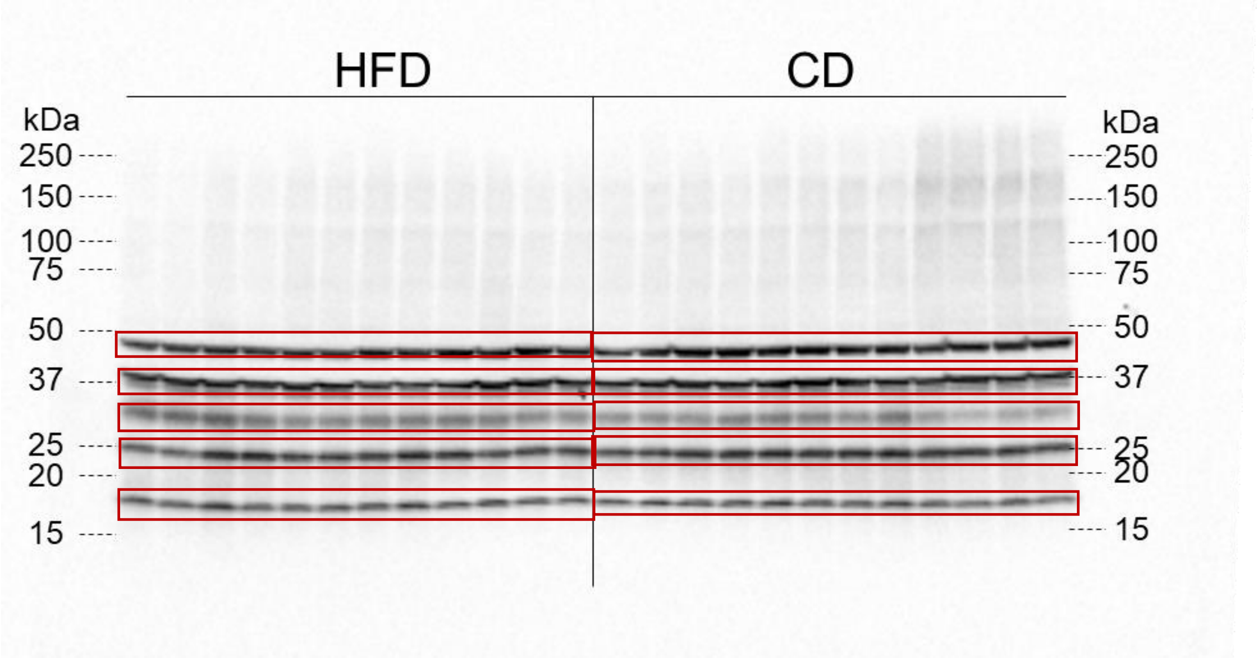

Blot #2 (days 1, 2, 7):

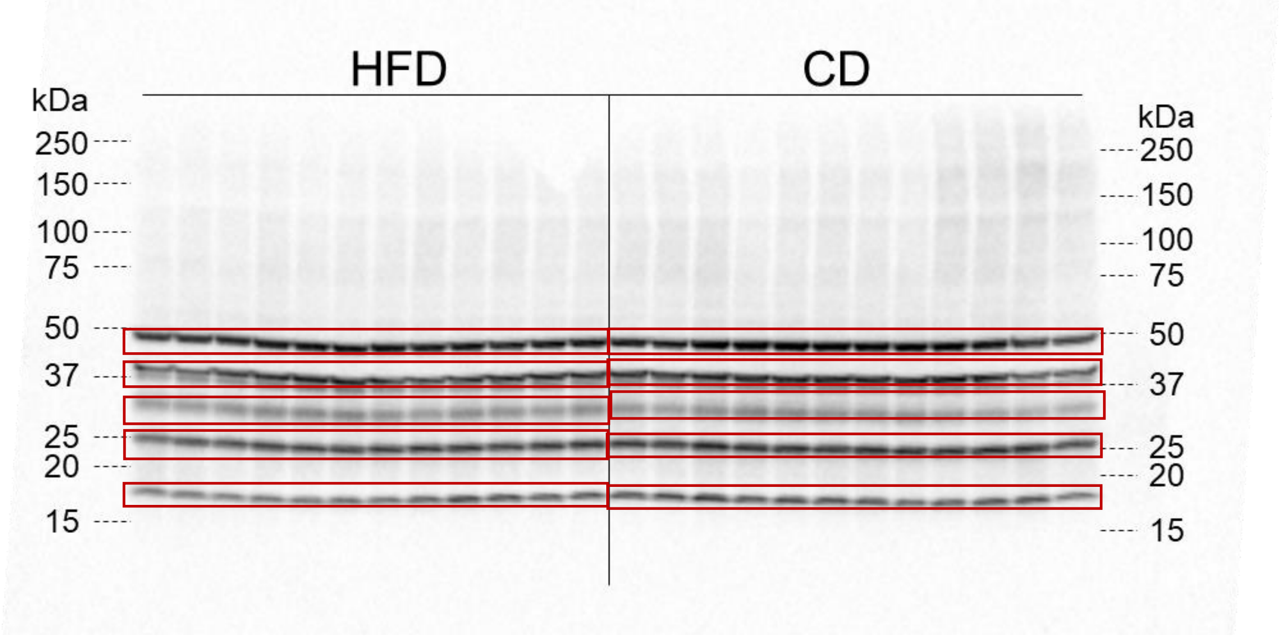

Note: A subset of this image is presented in figure 5E for representative purposes.

CERS1 – Isolated Mitochondria

Primary antibody: Ceramide synthase 1 (CERS1/LASS1), anti-rabbit polyclonal, Sigma Aldrich SAB2104843; 1:1,000 dilution
Secondary antibody: HRP-linked anti-rabbit IgG, Cell Signaling #7074S, 1:5,000 dilution

Blot #1 (day 0, 4, 12):

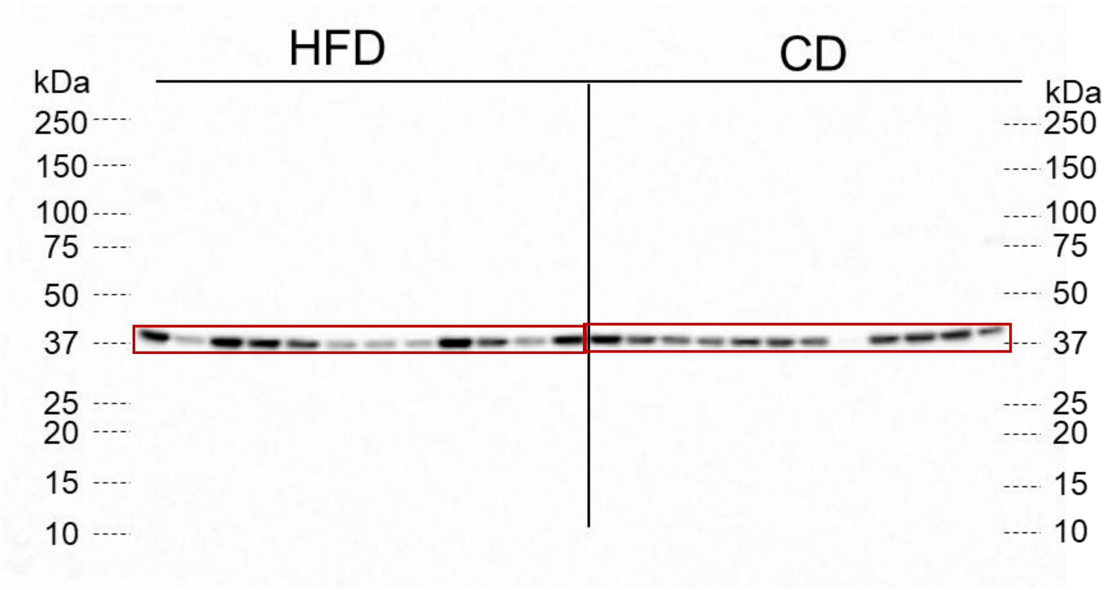

Blot #2 (day 1, 2, 7):

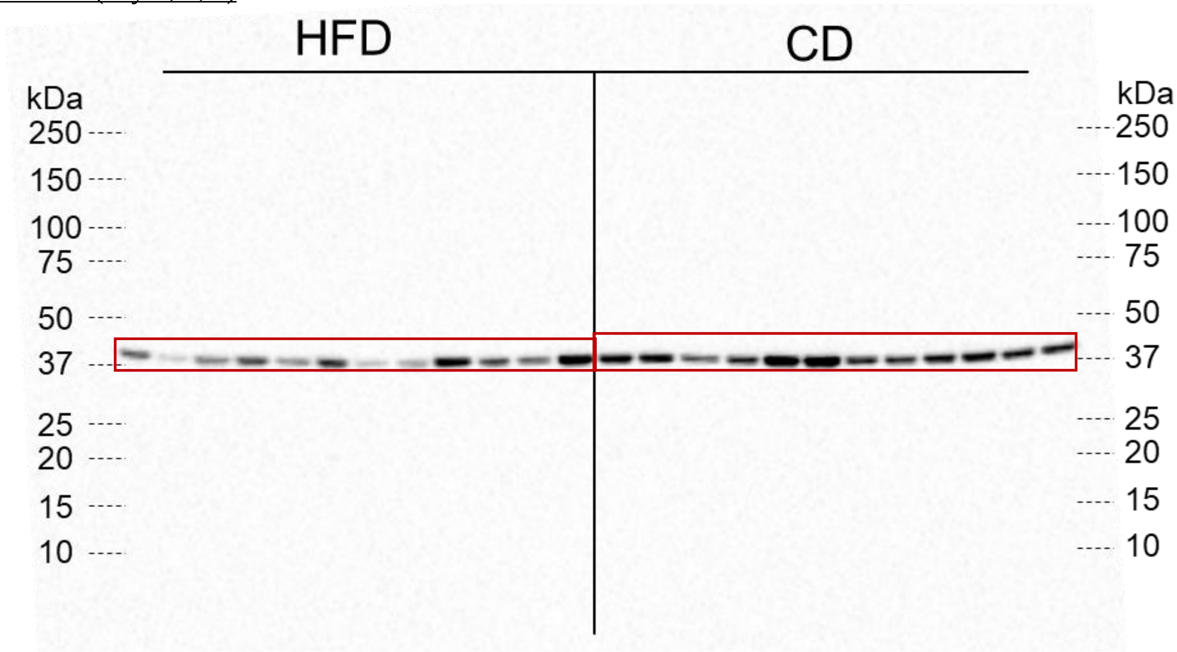

Note: A subset of this image is presented in figure S6B for representative purposes.

CERS2 – Isolated Mitochondria

Primary antibody: Ceramide synthase 2 (CERS2/LASS2), anti-mouse monoclonal IgG_1, Santa Cruz #390745; 1:1,000 dilution
Secondary antibody: HRP-linked anti-mouse IgG, Cell Signaling #7076S, 1:5,000 dilution

Blot #1 (day 0, 4, 12):

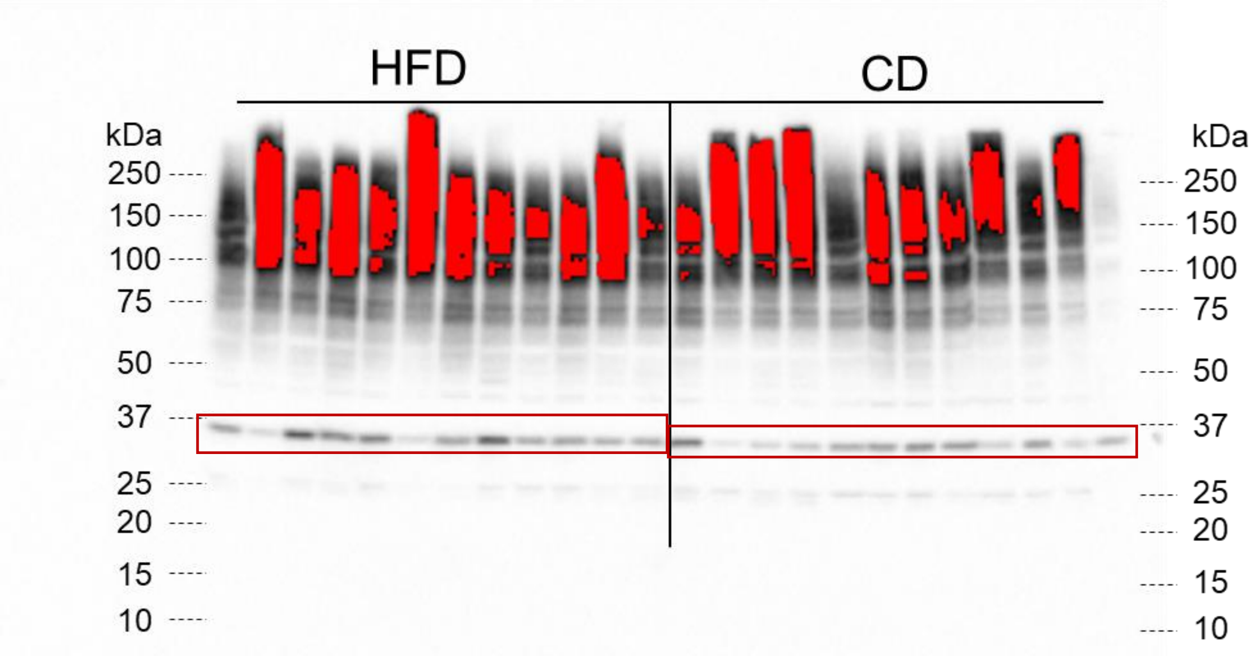

Blot #2 (day 1, 2, 7):

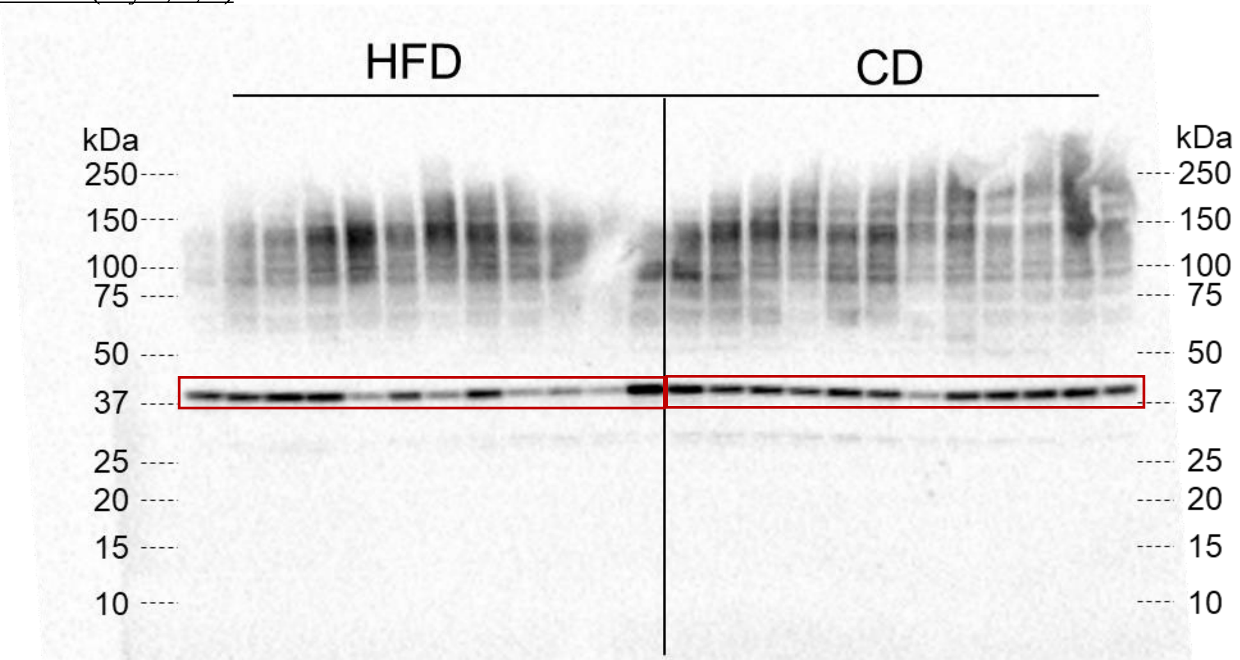

Note: A subset of this image is presented in figure S6B for representative purposes.

CERS6 – Isolated Mitochondria

Primary antibody: Ceramide synthase 6 (CERS6/LASS6), anti-mouse monoclonal IgG_{2a}, Santa Cruz #100554; 1:1,000 dilution
Secondary antibody: HRP-linked anti-mouse IgG, Cell Signaling #7076S, 1:5,000 dilution

Blot #1 (day 0, 4, 12):

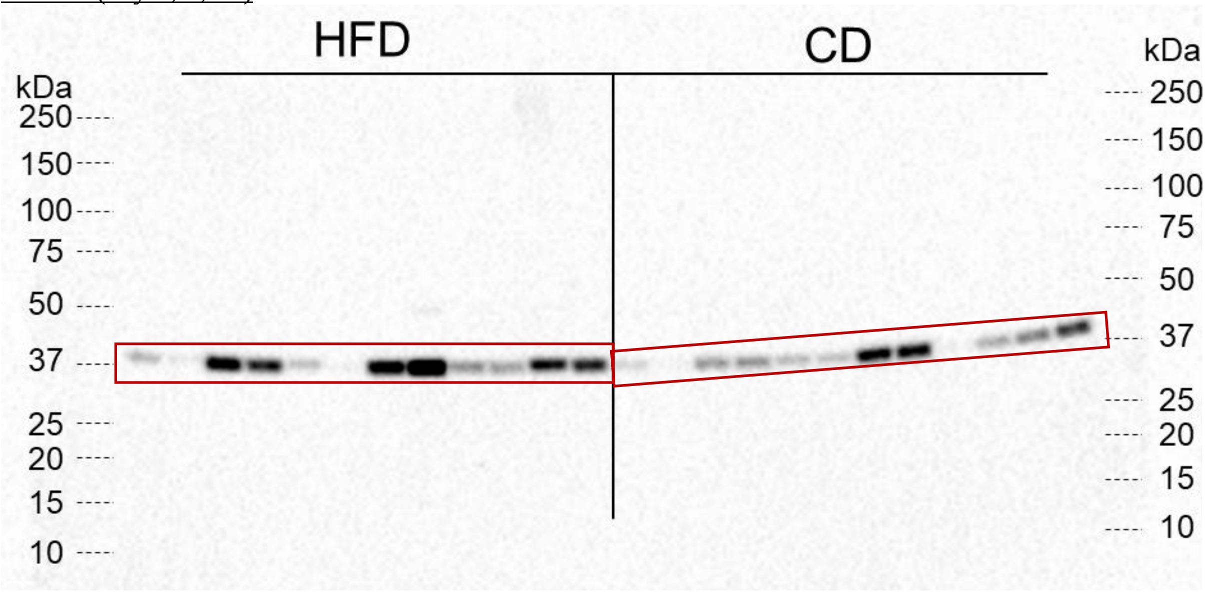

Blot #2 (day 1, 2, 7):

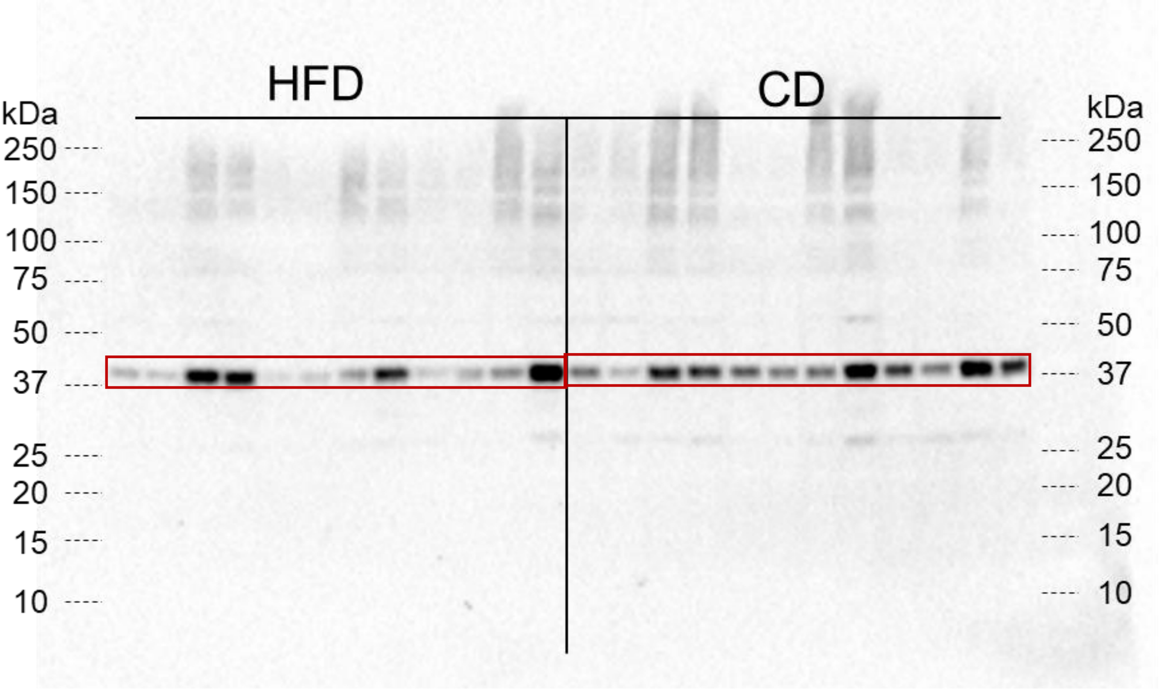

Note: A subset of this image is presented in figure S6B for representative purposes.

Organelle Detection – Isolated Mitochondria

Primary antibody: Organelle Detection Western Blot Cocktail, AbCam #133989; 1:1,000 dilution
Secondary antibody: HRP-linked anti-mouse IgG, Cell Signaling #7076S, 1:5,000 dilution

Blot #1 (day 0, 4, 12):

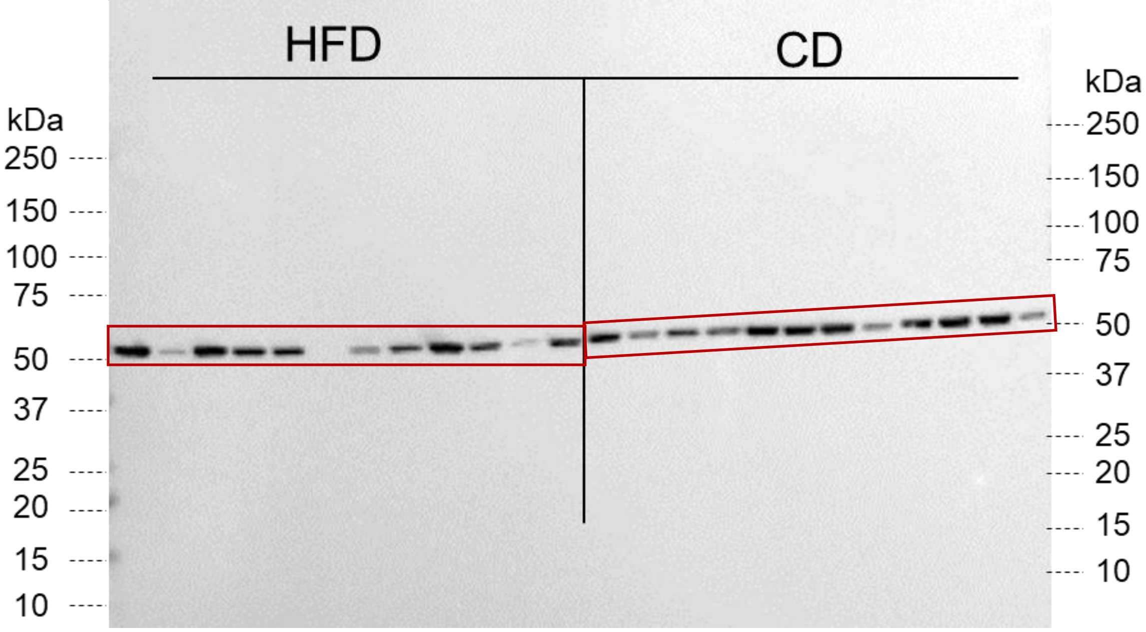

Blot #2 (day 1, 2, 7):

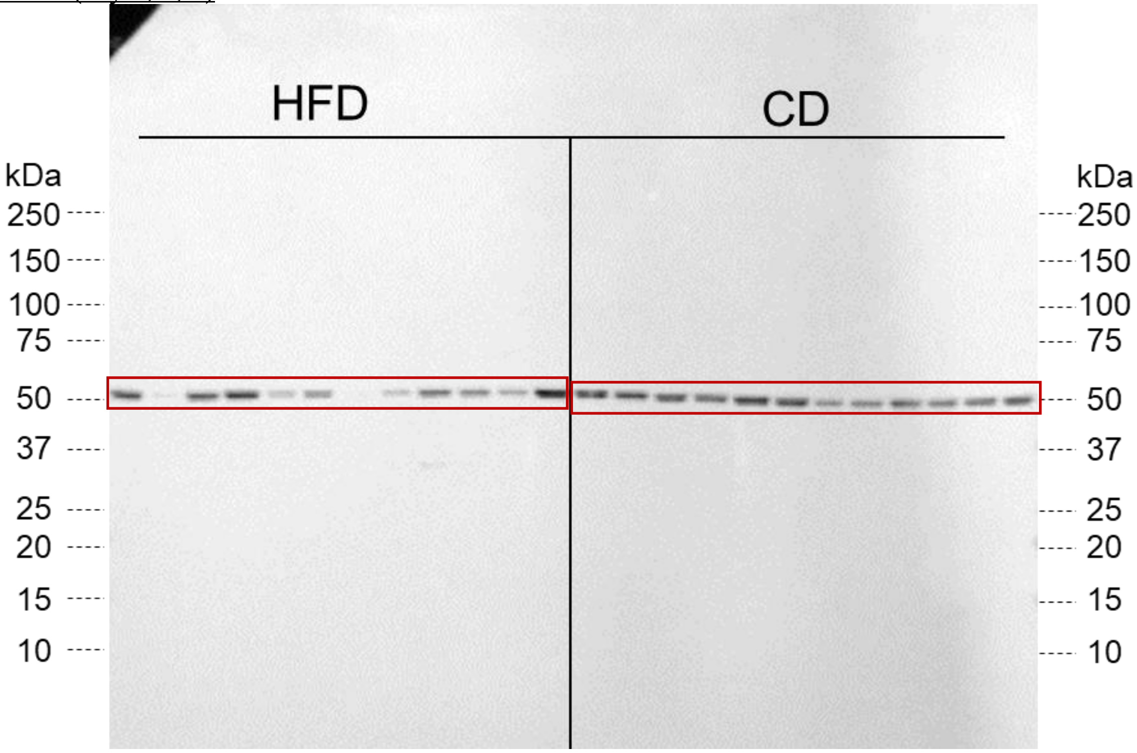

Note: A subset of this image is presented in figure S6C for representative purposes.

SUPPLEMENTAL REFERENCES

13. Chaurasia B, Tippetts TS, Monibas RM, Liu J, Li Y, Wang L, et al. Targeting a ceramide double bond improves insulin resistance and hepatic steatosis. *Science*. 2019;365(6451):386-92.

33. Chen Y, Berejnaia O, Liu J, Wang S-P, Daurio NA, Yin W, et al. Quantifying ceramide kinetics in vivo using stable isotope tracers and LC-MS/MS. *Am J Physiol Endo Metab*. 2018;315:E416-E24.

34. Zabielski P, Daniluk J, Hady HR, Markowski AR, Imierska M, Górski J, et al. The effect of high-fat diet and inhibition of ceramide production on insulin action in liver. *J Cell Physiol*. 2018.

35. Zabielski P, Błachnio-Zabielska AU, Wójcik B, Chabowski A, Górski J. Effect of plasma free fatty acid supply on the rate of ceramide synthesis in different muscle types in the rat. *PLoS One*. 2017;12:e0187136.

40. Berdyshev EV, Gorshkova I, Skobeleva A, Bittman R, Lu X, Dudek SM, et al. FTY720 inhibits ceramide synthases and up-regulates dihydrosphingosine 1-phosphate formation in human lung endothelial cells. *J Biol Chem*. 2009;284(9):5467-77.

41. Berdyshev EV, Gorshkova IA, Usatyuk P, Zhao Y, Saatian B, Hubbard W, et al. De novo biosynthesis of dihydrosphingosine-1-phosphate by sphingosine kinase 1 in mammalian cells. *Cell Signal*. 2006;18(10):1779-92.

42. Tserng KY, Griffin RL. Ceramide metabolite, not intact ceramide molecule, may be responsible for cellular toxicity. *Biochem J*. 2004;380(Pt 3):715-22.

61. Tserng K-Y, Griffin R. Quantitation and molecular species determination of diacylglycerols, phosphatidylcholines, ceramides, and sphingomyelins with gas chromatography. *Anal Biochem*. 2003;323:84-93.

62. Zabielski P, Hady HR, Chacinska M, Roszczyc K, Gorski J, Blachnio-Zabielska AU. The effect of high fat diet and metformin treatment on liver lipids accumulation and their impact on insulin action. *Scientific Reports*. 2018;8(1):7249.
